# Feedback regulation between histone H3 lysine 18 lactylation and TROP2‐mediated glycolysis drives metastatic progression of colorectal cancer

**DOI:** 10.1002/ctm2.70562

**Published:** 2026-01-03

**Authors:** Weifeng Wang, Yuxiang Deng, Weihao Li, Ruowei Wang, Chi Zhou, Yanbo Xu, Jiahua He, Le' en Liao, Jin Lan, Long Yu, Da Kang, Weili Zhang, Qingjian Ou, Zhizhong Pan, Yujing Fang, Peirong Ding, Junzhong Lin, Jianhong Peng

**Affiliations:** ^1^ Department of Colorectal Surgery Sun Yat‐Sen University Cancer Center, State Key Laboratory of Oncology in South China, Guangdong Provincial Clinical Research Center for Cancer Guangzhou Guangdong China; ^2^ Department of Thyroid and Breast Surgery Shenzhen Second People's Hospital The First Affiliated Hospital of Shenzhen University Shenzhen China; ^3^ Department of Thyroid Surgery Zhujiang Hospital of Southern Medical University Guangzhou Guangdong China

**Keywords:** colorectal cancer liver metastasis, glycolysis, H3K18la, lactate, TROP2

## Abstract

**Background:**

TROP2, a critical cell surface oncogenic signal transducer, is increasingly linked to refractory metastatic colorectal cancer (CRC) and other solid tumours. Robust lactate accumulation within metastatic niches correlates with pathological metastatic progression. Anti‐TROP2 antibody‐drug conjugates (ADCs) are clinically available but show limited efficacy in advanced metastatic CRC. Elucidating how TROP2 signalling orchestrates molecular and cellular programs enabling CRC metastatic progression would help improve metastasis therapies.

**Methods:**

Tissue microarray, immunohistochemistry, and western blotting delineated TROP2's pathological role in CRC liver metastasis (CRLM). Metabolomics characterised TROP2‐mediated metabolic effect. Western blot detected TROP2 responsive lactylation sites. Cell‐derived xenograft (CDX), intra‐splenic injection models, and patient‐derived xenografts (PDX) validated TROP2 or TROP2‐induced H3K18 lactylation (H3K18la) in CRLM pathogenesis and Acriflavine therapeutic response. Genome‐wide H3K18la profiling was performed by ChIP‐seq.

**Results:**

Here, we identify a self‐reinforcing positive feedback loop between H3K18la and TROP2 in CRC cells that drives CRC metastatic progression. We show that TROP2 is elevated during CRC metastatic process, with high TROP2 levels in liver metastases predicting increased post‐therapy recurrence in two distinct cohorts. We find that H3K18la levels are upregulated in CRC cells in response to TROP2 expression level. TROP2 promotes robust lactate production via the YBX1‐HIF‐1α signal axis. Targeting glycolytic flux decreases H3K18 lactylation and curbs TROP2‐driven CRLM colonisation and progression. Mechanistically, ChIP‐seq detection reveals H3K18la deposition at a set of pro‐metastatic gene promoters, promoting their expression. Crucially, TROP2‐induced H3K18la is found in turn sustaining TROP2 expression, forming a positive feedback loop that further accelerated metastatic progression. Pharmacologic HIF‐1α inhibition with acriflavine, an old FDA‐approved agent, suppresses TROP2‐high CRLM progression in multiple pre‐clinical models.

**Conclusions:**

Collectively, we establish H3K18la as a crucial epigenetic driver of TROP2‐mediated CRLM progression and propose that disrupting the H3K18la–TROP2 feedback loop offers a novel therapeutic strategy against CRC metastasis.

**Key points:**

- H3K18la is specifically increased in CRC cells in response to TROP2 signalling and drives TROP2‐mediated CRLM progression
- Genome‐wide analysis shows H3K18la deposition at the promoters of metastasis‐promoting genes drives their expression in TROP2‐high CRC.
- Lactate sustains TROP2 expression in CRC cells via H3K18la
- Acriflavine suppresses TROP2‐driven CRLM by targeting the H3K18la/TROP2 feedback loop

## Graphical abstract

- TROP2 is upregulated in CRC metastasis and promotes lactate overproduction via YBX1‐HIF‐1α signalling.
- TROP2 signalling induces H3K18la at metastasis genes promoters in CRC and promotes their expression.
- AccLactate sustains TROP2 expression in CRC cells via H3K18la
- Acriflavine suppresses TROP2‐driven CRLM by targeting the H3K18la/TROP2 feedback loop

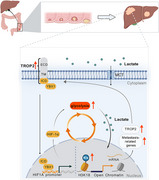

## 1 BACKGROUND

Colorectal cancer (CRC) represents the third most common malignancy and a leading cause of cancer‐related mortality worldwide.^1^, ^2^ Metastasis is the primary contributor to this mortality. In CRC, the liver serves as the primary metastatic organ, with nearly 50% of patients developing liver metastases at some point during disease progression.^3^, ^4^ For patients with colorectal liver metastasis (CRLM), radical liver resection may offer the potential for long‐term survival in roughly 50% of cases.^5^, ^6^ However, over 60% of patients experience relapses during follow‐up after the initial resection and systemic treatment.^7^, ^8^ Despite advances in immunotherapy, only about 5% of metastatic CRC cases benefit from immune checkpoint blockade (ICB), whereas the remaining 95% of patients have a poor prognosis with a 5‐year survival rate of less than 20%,^9^, ^10^ which existed as a progressive disease with no curative treatment options once metastasis occurred. Therefore, understanding the mechanism responsible for refractory metastatic progression remains the top challenge in CRC therapy and helps to identify novel targets.

Trophoblast surface antigen 2 (TROP2/TACSTD2), also known as gastrointestinal tumour‐associated antigen (GA7331), is a type I cell surface receptor glycoprotein composed of an extracellular domain, a single transmembrane helix, and a short cytoplasmic tail with a phosphatidylinositol 4,5‐bisphosphate (PIP2)‐binding motif, which is tightly regulated and limited in normal adult tissues and frequently overexpressed in multiple human carcinomas. TROP2 is an important regulator of oncogenic signalling that controls several key malignant processes in cancer cells, including proliferation, transformation, drug resistance and metastasis.^11^ While physiologically expressed in fetal intestinal progenitors,^12^ TROP2 is heterogeneously upregulated across CRC, with high TROP2 levels observed in a specific subset of cases.^13^, ^14^ Emerging evidence has highlighted a strong correlation between high TROP2 expression with refectory metastatic CRC and other solid tumours.^13^, ^15^, ^16^, ^17^ A recent study demonstrated that the released TROP2 extracellular domain (ECD) drives metastasis of prostate cancer cells.^18^ Notably, in human‐like mouse post‐resection CRC models, TROP2 is highly reactivated in cell populations responsible for liver metastatic colonisation and relapses following radical operation,^19^ positing TROP2 as the potential driver of refractory CRLM. Anti‐TROP2 antibody–drug conjugates (ADCs) are developed and used in clinical practice, with TROP2 as a transport gate for cytotoxic agents into cells.^20^ However, the specific role of TROP2 itself in CRC progression is still unclear and the efficacy of TROP2 ADCs in advanced and metastatic CRC is limited in clinical trials.^21^ Elucidating the mechanisms underlying how TROP2 expression and signalling modulates the molecular and cellular programs of CRC cells and promotes their metastatic progression would help to improve CRC metastasis therapies.

Previous research has suggested that CRC cells rewire metabolic pathways to adapt to metabolic stress inherent to the tumour microenvironment, thereby facilitating cancer metastasis.^22^, ^23^, ^24^ CRC cells rely on highly active glycolysis – commonly known as the Warburg effect – to sustain survival and colonisation in diverse metastatic niches, especially in the liver, the primary target organ of CRC which features a unique hypoxemic portal venous system.^25^, ^26^, ^27^ It is known that TROP2 modulates AKT signalling,^28^ which potentially supports glycolysis; However, whether CRC cells’ glycolytic activity directly responds to TROP2 expression and how it affects TROP2‐induced CRC metastatic behaviours remains unclear. Robust glycolysis leads to lactate accumulation in CRC cells. While previously defined as a metabolic waste product, lactate has recently emerged as a critical regulator of diverse cellular programs, including macrophage behaviours,^29^ oncogenesis,^30^ neovascularisation.^31^ Nevertheless, the role of accumulated lactate within metastatic microenvironment in CRC cell metastatic colonisation and progression remains elusive.

Growing evidence points out the fundamental role of histone in regulating chromatin architecture and function.^32^ Multiple epigenetic modifications, including methylation,^33^ acetylation,^34^ are powerful regulators of histone function and affect cellular processes. Lactate, derived from glycolysis within tumour, can initiate histone lysine lactylation (Kla) as a new modification that regulates chromatin accessibility and affects gene transcription programs. Our studies and others have reported that histone lactylation modulates diverse vital cellular and pathological processes including autophagy, tumour initiation, angiogenesis, immune escape and affects disease development.^35^, ^36^, ^37^, ^38^ However, whether glycolysis and lactylation directly responds to TROP2 oncogenic signal and modulate TROP2‐mediated CRC metastatic colonisation and progression remains unknown.

In this study, we aimed to investigate the role of glycolysis and accumulated lactate within metastatic environment in TROP2‐mediated CRC progression and liver metastasis. We demonstrated elevated TROP2 during CRC metastatic process, with high TROP2 levels in liver metastases predicting increased post‐therapy recurrence in two distinct cohorts. TROP2 promoted lactate overproduction via the YBX1‐HIF‐1α axis in CRC cells. Enhanced glycolysis and lactate induced histone H3 lysine 18 lactylation (H3K18la) in response to TROP2. Inhibiting lactylation effectively suppressed TROP2‐driven CRLM progression. Mechanistically, genome‐wide ChIP‐seq revealed H3K18la enrichment at promoters of a set of pro‐metastatic genes in TROP2 high CRC, promoting their expression. Crucially, TROP2‐induced H3K18la in turn sustained TROP2 expression, constituting a positive feedback loop that further accelerated CRC metastatic progression. Pharmacologic HIF‐1α inhibition with acriflavine suppressed TROP2‐high CRLM in splenic injection and patient‐derived xenograft (PDX) pre‐clinical models. Collectively, these findings link liver metastatic progression to lactylation within the metastatic niche and uncover a TROP2/H3K18la feedforward loop, suggesting potential therapeutic strategies for TROP2‐high CRC metastatic progression.

## 2 METHODS

### 2.1 Cell lines and cell cultures

Human CRC cell lines (SW480, DLD‐1, Lovo, HT‐29, RKO, SW620, Caco2, HCT116), mouse CRC cells (MC38), normal colon epithelial cells (CCD‐841 CoN), and HEK293T cells were purchased and cultured in complete medium with antibiotics and 10% FBS at 37°C, 5% CO_2_. Each cell line was STR‐verified for identity and confirmed mycoplasma‐free prior to use. Detailed information and Research Resource Identifiers (RRIDs) on the antibodies, chemical reagents, commercial assays and experimental models used in this study are provided in Table S1.

### 2.2 Patients and specimens

Between June 2001 and December 2016, we collected 149 colorectal liver oligometastasis (CLO; defined as ≤5 liver metastases confined to the liver^39^) tissue specimens (primary and liver metastatic) from surgical samples at the Sun Yat‐sen University Cancer Center (SYSUCC). Eligibility required (i) pathological proof of colorectal adenocarcinoma, (ii) one isolated hepatic lesion, (iii) no distant disease beyond the liver before surgery, (iv) simultaneous R0 removal of primary tumour and liver deposit, and (v) at least 36 months of postoperative follow‐up. Additionally, from September 2012 to September 2018, we obtained tissue specimens from 110 CRLM patients (non‐oligometastatic) at SYSUCC. The detailed clinical information of both clinical cohorts are described in Table S2. The management approach and feasibility of liver metastasis treatment for each patient were determined by consensus of the multidisciplinary team (MDT). Metastatic recurrence or progression status was assessed at baseline and every 6 weeks after operation using abdominal/pelvic/chest CT or MRI. Informed consent for the use of imaging and clinical data was obtained from the patients. The study was approved by the Institutional Research Ethics Committees of Sun Yat‐sen University Cancer Center (Guangzhou, China, approval number: GZR2020‐071).

### 2.3 Cell transfection and lentivirus

Commercial shRNA#1, shRNA#2 or overexpression lentiviral particles targeting TROP2 were purchased from Genecopoeia (America) and used to infect target cells for 2 days. Stable CRC cell lines were established by puromycin selection (2 µg/mL) for 10 days. TROP2 overexpression and truncation plasmids were constructed by Kidan Biosciences (China), while YBX1‐targeting siRNAs were synthesised by RiboBio (China). All constructs were transfected into cells using jetPRIME® transfection reagent (Polyplus, 101000046) following manufacturer‐optimised protocols.

### 2.4 Tissue microarray analysis

The detailed clinical information of tissue microarray detection cohorts are described in Table S3. Total RNA was extracted from formalin‐fixed, paraffin‐embedded (FFPE) tissue sections or fresh‐frozen specimens using the RNeasy Mini Kit (Qiagen), followed by quality assessment via Agilent Bioanalyzer 2100 (RNA Integrity Number ≥7.0). For Human Transcriptome Array (HTA) 2.0 analysis, 100 ng of RNA was amplified and biotin‐labelled using the Affymetrix WT PLUS Reagent Kit, adhering to the manufacturer's protocol. Labelled cDNA was hybridised to the HTA 2.0 arrays (Thermo Fisher Scientific). Hybridisation (45°C, 16 h; GeneChip Hybridization Oven 645) was followed by washing/staining (Affymetrix Fluidics Station 450). Array scanning (GeneChip Scanner 3000 7G) generated raw data processed via Affymetrix Expression Console v1.4 using the Robust Multi‐array Average (RMA) normalisation. Quality control parameters (scaling factors, > 90% present calls, RNA degradation profiles) were validated. Differential expression analysis (limma R package: FDR < 0.05, |FC| > 1.5) preceded pathway enrichment.

### 2.5 Western blot analysis

Tissues and cells were treated with RIPA buffer and then centrifuged at 4°C with a force of 13 000 × *g* for 30 min. The detailed clinical information of patients with fresh tumour tissue samples for Western blot or PDX construction are described in Table S4. Protein lysates, in equal measures, underwent 10% SDS‐PAGE and were transferred onto PVDF membranes. These were subsequently blocked with a solution of 5% BSA in TBS with 0.1% Tween‐20 for 1 h, followed by an overnight incubation at 4°C with specific primary antibodies. The primary antibodies utilised encompassed a range of targets including anti‐TROP2, anti‐p‐YBX1 (ser102), anti‐YBX1, anti‐PDK1, anti‐PFKFB3, anti‐PKM2, anti‐PanKla, anti‐H3K18la, anti‐H3K9la, anti‐H4K8la, anti‐H4K12la, anti‐H4K5la, anti‐HIF‐1α, anti‐vinculin, anti‐Lamin A, anti‐LDHA, anti‐FLAG tag, anti‐HA tag, anti‐MCT4, anti‐EP300. After washing, the membranes were incubated for 1 h at room temperature with corresponding HRP‐conjugated secondary antibodies.

### 2.6 Immunohistochemistry

Immunohistochemical (IHC) examination on formalin‐fixed, paraffin‐embedded CRC/CRLM tissues followed the established procedure. The primary antibodies employed for IHC included anti‐TROP2, anti‐YBX1, anti‐HIF‐1α, anti‐PDK1, anti‐PFKFB3, anti‐Pan Kla, anti‐MKI67, anti‐Cleaved CASP3, anti‐CD3, anti‐CD8, anti‐Foxp3, anti‐CD206 and anti‐H3K18la. The assessment of IHC staining was conducted by two independent pathologists specialising in gastrointestinal diseases, who were unaware of the patients' details and clinical outcomes. The IHC scoring system took into account the proportion and intensity of stained cells. The proportion score, which indicated the percentage of positively stained cells, was graded from 0 to 4 (0 for 0%–5%, 1 for 5%–25%, 2 for 26%–50%, 3 for 51%–75%, and 4 for 76%–100%). The staining intensity was graded as 0 (no staining), 1 (mild), 2 (moderate), or 3 (intense). The Cutoff Finder tool was utilised to establish the optimal threshold for determining high or low expression levels of TROP2, H3K18la.

### 2.7 Tumour implantation and re‐isolation

CRC cell line‐based xenograft (CDX) models were performed. Among them, NOD.SCID mice (GemPharmatech, China) were used to construct Lovo‐based CDX models. When tumour diameters exceeded 2 cm, the mice were euthanised, and the tumours were harvested. All experimental procedures adhered to the National Institutes of Health Guide for the Care and Use of Laboratory Animals (NIH Publications No. 8023, revised 1978) and were approved by the Animal Care and Use Committee of Sun Yat‐sen University (L102022023001K, Guangzhou, China). Lovo CDX were disaggregated into single cells using collagenase IV/DNase I digestion and passed through 70‐µm strainers. Differential centrifugation (300 × *g*, 5 min) removed debris, followed by ACK lysis to eliminate red blood cells. Cancer cells were enriched by Percoll density gradient centrifugation (30%/70%, 400 × *g*, 20 min). The final purity (> 90% EpCAM+/CD45–) was confirmed by flow cytometry, and cell viability was assessed by trypan blue exclusion.

### 2.8 Sample preparation of cell lysate for metabolomic analyses

Suspension cells (10^7^ cells/sample) were quenched with 4× ice‐cold 150 mM NaCl, followed by centrifugation (3000 × *g*, 5 min, 4°C) to pellet cells after supernatant removal. For adherent cells, monolayers were washed thrice with ice‐cold NaCl, detached via scraping in chilled solution, and pelleted identically. Pellets were subjected to modified Bligh‐Dyer extraction (methanol/chloroform/3.8 mM tricine, 1:1:0.5) to isolate aqueous polar metabolites. The aqueous phase was stored at −80°C, vacuum‐dried at 4°C (CentriVap, Labconco), and reconstituted in 5% methanol‐water prior to LC‐MS analysis.

### 2.9 Metabolomic and lipidomic analysis by UPLC‑MS

Metabolites and lipids were analysed using an ACQUITY UPLC I‑Class system (Waters) coupled to an Orbitrap mass spectrometer (Thermo Fisher). Full‑scan spectra (70 000 resolution) and dd‑MS^2^ scans (35 000 resolution) were acquired in dual ESI polarity. Polar metabolites were separated on an HSS T3 column with 0.1% formic acid in water (A) and acetonitrile (B). Lipids were profiled on a BEH C8 column using ammonium acetate/acetic acid in water (A) and acetonitrile‑isopropanol (7:3) with the same additive (B). QC included randomised sequences, pooled QC injections, and triplicate runs (4 µL each).

### 2.10 Quantification of lactate

Lactate levels were measured using the Lactate Assay Kit‐WST (DOJINDO) according to the manufacturer's instructions.

### 2.11 ECAR/OCAR measurement

Extracellular acidification rate (ECAR)/oxygen consumption rate (OCR) measurements were conducted using an XF96 Extracellular Flux Analyzer from Seahorse Bioscience, following a previously described methodology.^40^ For ECAR, DLD‐1 and SW480 adherent cells were seeded into XF96 (V3) polystyrene cell culture plates and incubated overnight in a humidified incubator at 37°C without CO_2_ after adding 200 µL of Seahorse XF Calibrant Solution to each well. The instrument was pre‐warmed for 2 h prior to measurements. Glutamine‐based test medium was prepared, and cells were incubated with test medium for 1 h. Glucose, Oligomycin, and 2‐DG were sequentially added. All experimental procedures were carried out at 37°C. The ECAR data points were recorded as the individual rates measured in each well during assay cycles and are expressed as absolute rates (mpH/min). The ECAR data were normalised to protein concentration using the BCA assay. For OCR, the same procedure was followed, with Oligomycin, FCCP, and Antimycin A/Rotenone added. OCR values were also normalised to protein concentration.

### 2.12 Immunofluorescent staining

Cells that had attached to the surface were first fixed using methanol and then carefully rinsed with PBS on two occasions. Subsequently, the cells were treated with a permeabilisation buffer (eBioscience, 00‐8333‐56) for a duration of 10 min at a chilled temperature of 4°C. Once the cells were blocked with a solution containing 5% BSA in TBS/Tween‐20 for an hour at ambient temperature, they were exposed to primary antibodies specific for TROP2, YBX1, HIF‐1αunder cold conditions overnight at 4°C. Fluorescent‐tagged secondary antibodies were added in a light‐protected environment, followed by the application of DAPI for nuclear visualisation. The subsequent imaging was performed utilising an Olympus® Fluoview FV1000 microscope (Japan), with the assistance of the FV10‐ASW 4.0 software to dissect the multispectral images.

### 2.13 Cell proliferation assays

Cells were plated in 96‐well plates with a density of 2000 cells per well for spheroid cells and 500 cells per well for adherent cells. Cell viability, normalised to the initial mean viability at day 0, was determined every 2 days using the CCK8 (DOJINDO, CCK8) assay.

### 2.14 Colony formation

Cells were seeded in 6‐well plates (1000 cells per well) with 3 replicates and were then cultured with complete medium at 37°C with 5% CO_2_. The resulting colonies were visualised with crystal violet staining and enumerated after a period of approximately two weeks.

### 2.15 Transwell assays

Initially, 10^5^ cells suspended in 400 µL of serum‐free medium were placed into Transwell inserts (Corning), which were then positioned in a 24‐well plate. Subsequently, 600 µL of medium with 20% FBS was added to the lower compartment. Following a 24‐h incubation, the cells that had migrated were fixed with methanol and stained with crystal violet. For invasion assessments, 50 µL of serum‐free medium with 10% Matrigel was applied to the upper chamber prior to cell seeding. The cells that migrated or invaded were photographed and quantified using a microscope at a 20× magnification.

### 2.16 Coimmunoprecipitation

Co‐IP was performed using the Pierce™ Classic Magnetic IP/Co‐IP Kit (Thermo, 88804) according to the manufacturer's protocol. Cells were lysed on ice in lysis buffer (50 mM HEPES, pH 7.4, 150 mM NaCl, 1% Triton X‐100, 10% glycerol, 2 mM MgCl_2_, 2 mM EGTA) supplemented with protease and phosphatase inhibitor cocktail (Thermo, 78446). The lysates were centrifuged to remove insoluble materials, and antibodies were incubated with beads overnight at 4°C. Following the wash steps, proteins retained on the membrane were visualised via Western blot analysis.

### 2.17 IP/MS

Both HEK293 and DLD‐1 cells were transfected with TROP2 in advance. Then IP experiment was performed as described above. The separated protein was subsequently prepared for MS analysis.

### 2.18 Chromatin immunoprecipitation sequencing

The ChIP assay was performed using the Pierce™ Magnetic ChIP Kit (Thermo, 26157) according to the manufacturer's instructions. After cross‐linking and chromatin digestion, the digested chromatin was incubated with 5 µg of antibody (anti‐H3K18la‐ChIP Grade, PTM‐1427RM, or anti‐IgG, #3900) overnight at 4°C. ChIP‐grade Protein A/G magnetic beads (Thermo, 26157) were added the next day and incubated for an additional 4 h. Purified DNA fragments were then used to construct ChIP‐seq libraries and sequenced on the HiSeq 2500 platform (Illumina). Anti‐YBX1‐ChIP grade (ab76149) is used for ChIP‐qPCR assay. The primers used for qPCR analysis are listed in Table S5.

### 2.19 RNA isolation, quantitative real‐time PCR (RT‐qPCR)

RNA was extracted with TRIzol and checked on a Nanodrop (260/280 ≥ 1.8, 260/230 ≥ 2.0), reverse‐transcribed with the PrimeScript RT Kit, and quantified by SYBR Green qPCR on a LightCycler 480 II.

### 2.20 Spleen injection CRLM and PDX models

For spleen injection model construction, C57BL/6 mice were randomised into experimental groups (*n* = 5/group) and underwent intrasplenic injection of MC38 cells (2.0 × 10⁶ cells in 100 µL) stably overexpressing TROP2 or negative control (NC) using insulin syringes, with splenic compression applied for 3 min to ensure haemostasis. Starting 14 days post‐implantation, intraperitoneal treatments were administered as follows: 2‐deoxy‐D‐glucose (2‐DG, 500 mg/kg twice weekly), acriflavine (10 mg/kg daily), or placebo. Body weights were monitored every 3 days until scheduled euthanasia on day 25, as determined by preliminary kinetic studies. Livers were harvested, weighed, and photographed for macroscopic metastatic burden assessment. Histopathological analysis included haematoxylin and eosin (H&E)‐stained serial sections to quantify metastatic foci. All experimental procedures adhered to blinded randomisation protocols for treatment allocation and outcome evaluation.

Immunocompromised NOD.SCID mice (GemPharmatech, China) were used to establish patient‐derived xenograft models (PDX). Fresh colorectal cancer (CRC) specimens were subcutaneously engrafted into mice (F0 generation) under sterile conditions. Upon reaching the appropriate tumour volume, F0 xenografts were aseptically dissected into uniform fragments for subsequent F1 generation engraftment. Tumour‐bearing F1 mice were blindly randomised into acriflavine therapeutic or placebo group (*n* = 5/group) when lesions became measurable (> 100 mm^3^). Tumour dimensions were recorded triweekly over 4–5 weeks using digital callipers, with volumes calculated as (length × width^2^ × 0.5). Terminal procedures involved surgical resection of tumours at study endpoint (days 28–35 post‐treatment initiation) for pharmacodynamic analyses. All protocols adhered to AAALAC‐accredited animal welfare standards.

### 2.21 Source of PI3K‐AKT signalling pathway genes

The PI3K‐AKT pathway gene set was curated by integrating two complementary resources: (1) The GSEA WP_PI3KAKT_SIGNALING_PATHWAY (source link: https://www.gsea‐msigdb.org/gsea/msigdb/human/geneset/WP_PI3KAKT_SIGNALING.html). (2)The PI3K/AKT Phosphorylation Substrates Table from Cell Signaling Technology (CST) (https://www.cellsignal.cn/learn‐and‐support/reference‐tables/pi3k‐akt‐substrates‐table). Both resources derive their phosphorylation site annotations and supporting references from the PhosphoSitePlus® database (www.phosphosite.org).

### 2.22 Induction of hypoxic conditions

To induce hypoxic conditions, cells were placed in a sterilised modular incubator chamber (hypoxia chamber). The chamber was flushed for 10–15 min with a pre‐mixed gas mixture containing 1% O_2_, 5% CO_2_, and balance N_2_ to complete air displacement. After flushing, the chamber was sealed and placed in a standard 37°C incubator for the designated duration of hypoxia exposure.

### 2.23 MeRIP‐qPCR and RNA stability assay

The m5c meRIP‐qPCR assay was performed according to the manufacturer's instructions (Bersinbio, Guangzhou, China). CRC cells were cultured in distinct media containing 5 µg/mL actinomycin D for 0, 4, and 8 h, respectively. The total RNAs were extracted and quantitative RT‐PCR was conducted.

### 2.24 Immune‐cell quantification

A minimum of three representative 200× magnification fields were examined per sample, and the final data are presented as the average cell count per field.

### 2.25 Statistics analysis

All data are expressed as mean ± standard error of the mean (SEM) unless otherwise described. Statistical analyses were performed using GraphPad Prism software (version 8.0) and the significance of differences was assessed by two tail unpaired Student's *t*‐test or one‐way or two‐way analysis of variance (ANOVA) followed by Sidak's multiple comparisons test. The statistical parameters can be found in the figures and figure legends. Significance was set as *p* ≤.05 and expressed as *p ≤.05, **p ≤.01, ***p ≤.001, ****p ≤.0001.

## 3 RESULTS

### 3.1 Elevated TROP2 expression in colorectal cancer liver metastases predicts post‐hepatectomy recurrence and poor prognosis

**FIGURE 1:**
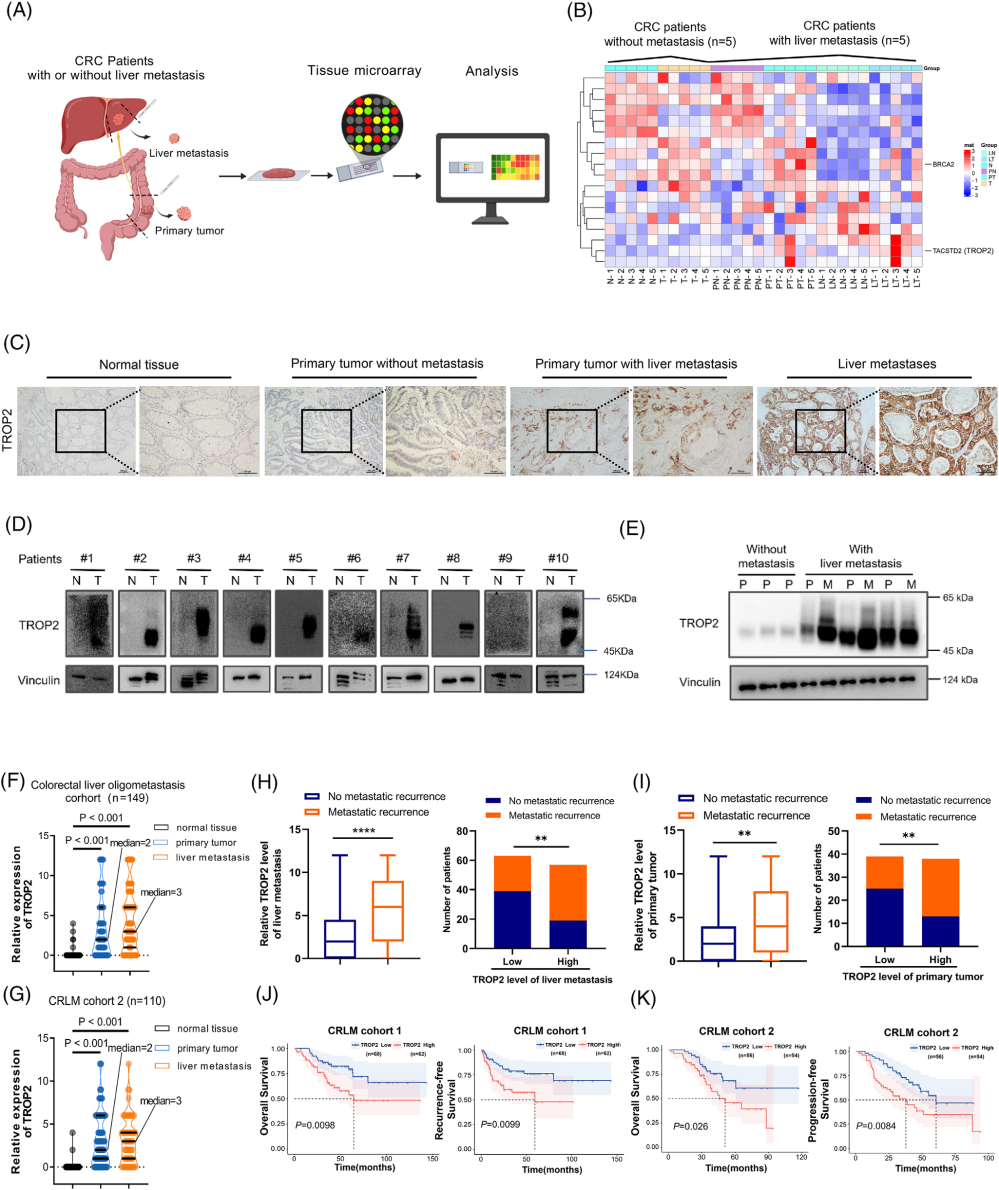
Elevated TROP2 expression in colorectal cancer liver metastases predicts post‐hepatectomy recurrence and poor prognosis. (A) Schematic diagram of tissue microarray analysis in colorectal cancer (CRC) patient samples. (B) Tissue microarray analysis depicting gene expression alterations in primary tumours from CRC patients without metastasis (*n* = 5; N, normal tissue; T, tumour) compared with primary tumours and liver metastases from CRLM patients (*n* = 5; PN, primary site normal tissue; PT, primary tumour; LN, normal liver tissue near metastasis; LT, liver metastasis). (C) Representative cases demonstrating high versus low TROP2 expression in matched primary tumours and liver metastases from CRLM patients (*n* = 1), primary tumours from CRC patients without metastasis (*n* = 1), and normal tissues, analysed by immunohistochemistry (IHC) staining (scale bar: 100 µm). (D) Western blotting detection of TROP2 in 10 matched CRC tissues (T) and adjacent noncancerous tissues (N). (E) Western blotting detection of TROP2 in primary tumours from CRC patients without metastasis (P) and matched primary tumours and liver metastases (P and M) from CRLM patients (*n* = 3). (F, G) Violent plot showing significantly high level of TROP2 in primary and liver metastasis sites of CRLM clinical cohorts compared to matched normal tissues, analysed by immunohistochemistry (IHC) staining; ****p ≤.0001. (F) CRLM cohort 1, Patients with colorectal liver oligometastasis (CLO) (*n* = 149). (G) CRLM cohort 2, CRLM patients with non‐oligometastasis (*n* = 110). (H) Statistics of relative TROP2 levels in liver metastasis of CLO patients with (*n* = 62) (right) or without (*n* = 61) (left) metastatic recurrence after curative tumour resection, and the number of CLO patients with low or high TROP2 levels in each group; **p ≤.01, ***p ≤.001, by Mann–Whitney test (left), or chi‐square test (right). (I) Relative TROP2 levels in primary tumours of CLO patients with (*n* = 39) (right) vs. without (*n* = 38) (left) metastatic recurrence post‐operation, and patient counts by primary TROP2 level; **p ≤.01, ***p ≤.001, as above. (J) Overall survival (left) or Recurrent‐free survival (right) Kaplan–Meier curve of 2 groups of patients based on TROP2 levels in CRLM cohort 1. (K) Overall survival (left) or progression‐free survival (right) Kaplan–Meier curve of 2 groups of patients in CRLM cohort 2.

Metastasis is a complex process involving proliferation, migration, circulation survival and liver colonisation in CRC cells that need multiple pro‐metastatic and pro‐survival signal activation.^41^ To identify the dependent regulators in this process, we profiled mRNA in fresh treatment‐naive CRC primary and liver‐metastatic surgical samples via tissue microarray (Figure 1A). TROP2 is one of the most powerful cell surface oncogenic signalling transductors. Microarray analysis revealed significantly elevated TROP2 transcription levels in CRLM and matched primary lesions compared to CRC cases without metastasis, as confirmed by a minimum follow‐up of three years (Figure 1B). Immunohistochemistry (IHC) staining further confirmed elevated TROP2 protein in CRLM, particularly within liver metastases (Figures 1C and S1D). Analysis of The Cancer Genome Atlas (TCGA) and the TNM plot database demonstrated a gradient increase of TROP2 with CRC stage (Figure S1A and B) and higher TROP2 in metastases versus primary tumours (Figure S1C). Importantly, in the liver metastatic cell lines KM12SM (spontaneous metastasis) and KM12L4 (experimental metastasis), generated by injecting the parental KM12C cell line into the spleen or cecum of mice to induce experimental and spontaneous liver metastasis, TROP2 was consistently markedly elevated compared to the parental KM12C cells (Figure S1E), which indicated TROP2's role in CRC liver metastatic capacity.^42^, ^43^ Western blotting confirmed heterogeneously increased TROP2 protein levels in CRC tumours compared to the matched adjacent normal tissues from 10 random CRC patients (Figure 1D). Increased TROP2 was consistently observed in both primary and liver metastatic tissues compared to those without metastasis using the western blot method (Figure 1E). We also found elevated TROP2 levels in CRC peritoneal and lung metastasis specimens (Figure S1I). We then analysed TROP2 expression in the CRC liver oligometastasis (CLO) cohort (CRLM cohort 1, initially resectable) and CRLM cohort 2, assessing its clinical significance. IHC staining showed markedly increased TROP2 levels in both primary and liver lesions in both cohorts (Figure 1F and G). In CLO cohort, high TROP2 levels in both liver metastase (Figure 1H) or primary lesion (Figure 1I) were associated with markedly increased metastatic recurrence rates after curative hepatectomy. High TROP2 level was associated with shorter overall survival (OS) and recurrence/progression‐free survival (RFS/PFS) in both cohorts (Figure 1J and K). Positive correlations were also observed between TROP2 expression in liver metastases /primary lesions and serum levels of carcinoembryonic antigen (CEA) (Figure S1F), Carbohydrate antigen 19‐9 (CA19‐9) (Figure S1G), and the matched MKI67 levels (Figure S1H). These data indicated that TROP2 is progressively elevated during CRC metastasis, with its high expression in liver metastases is linked to poor post‐treatment outcomes.

### 3.2 H3K18 lactylation is increased in CRC cells in response to TROP2 expression level

**FIGURE 2:**
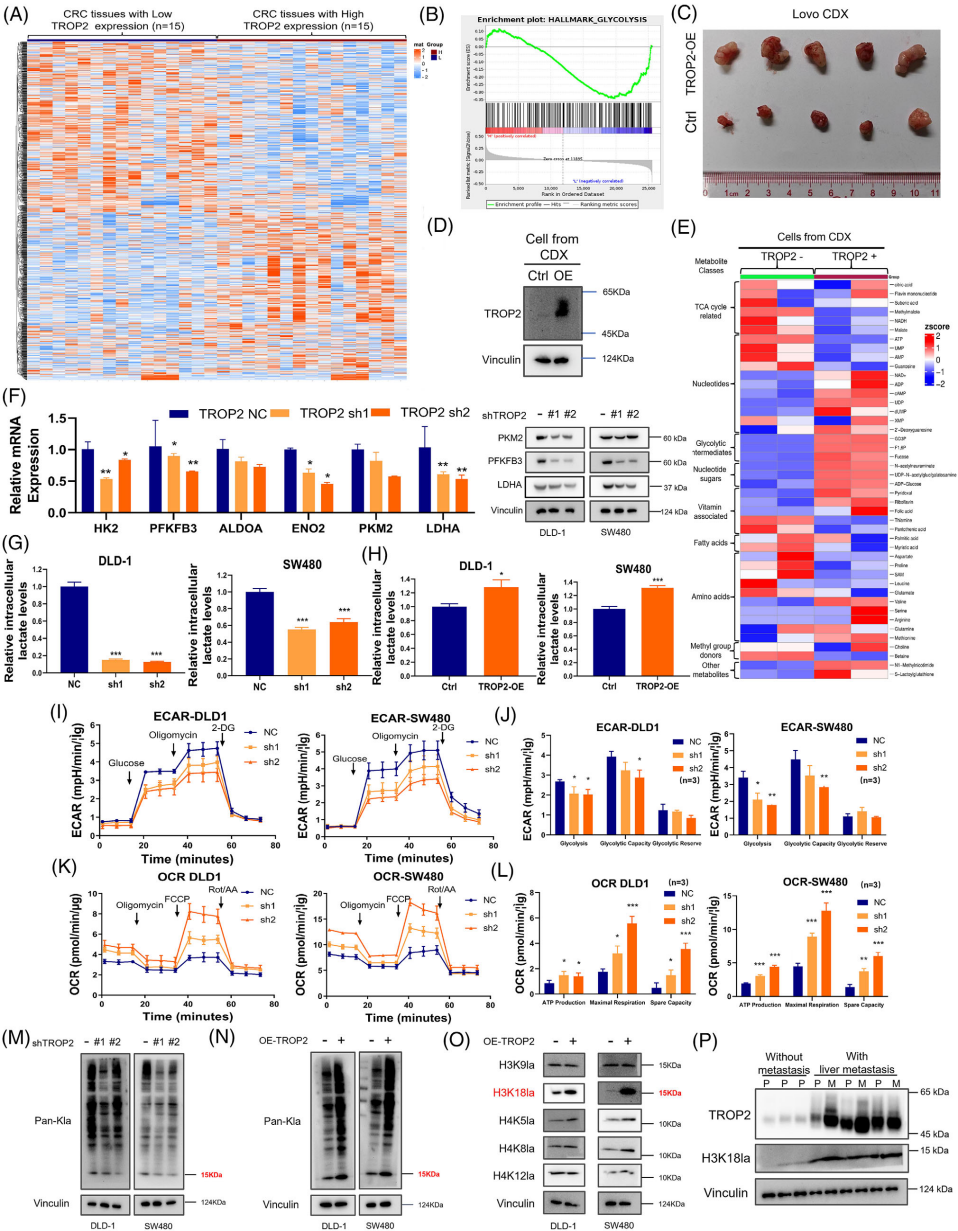
H3K18 lactylation is increased in CRC cells in response to TROP2 expression level. (A) Heatmap of 1556 differentially expressed genes in CRC tissues with low (blue) or high (brown) TROP2 expression, based on tissue microarray detection (803 downregulated and 753 upregulated in TROP2‐L versus TROP2‐H, all *p* ≤.05; *n* = 30; High, *n* = 15; Low, *n* = 15; based on median expression). (B) GSEA analysis demonstrating correlation of high TROP2 expression with Hallmark Glycolysis in CRC tissues, based on tissue microarray detection. (*n* = 30; High, *n* = 15; Low, *n* = 15; based on median expression). (C) Photographic comparison of Lovo CDX between groups. (D) Western blotting detection of TROP2 in re‐isolated cells from CDX of each group. (E) Metabolomic comparison of TROP2 positive and negative cells re‐isolated from two groups of CDX. Two biological replicates are shown as separate columns for each cell type. (F) Expression of glycolysis key enzymes was detected in SW480 cells following TROP2 silencing by RT‐qPCR and western blotting; *p ≤.05, **p ≤.01. (G, H) Abundance of intracellular lactate in DLD‐1 and SW480 cells following TROP2 silencing (G) or overexpression (H) as determined by a lactate colorimetric kit, normalised to abundance in NC (G) or Ctrl (H) group; *p ≤.05, ***p ≤.001. (I) Extracellular acidification rate (ECAR) levels of DLD‐1 and SW480 cells following TROP2 silencing. (J) Statistical analysis of changes in glycolysis, glycolytic capacity, and glycolytic reserve following TROP2 silencing, as assessed by ECAR detection; *p ≤.05, **p ≤.01. (K) Oxygen consumption rate (OCR) levels of DLD‐1 and SW480 cells following TROP2 silencing. (L) Statistical analysis of changes in ATP production, maximal respiration and spare capacity following TROP2 silencing, as assessed by OCR detection; *p ≤.05, **p ≤.01, ***p ≤.001. (M, N) Western blotting detection of Pan‐Kla levels in DLD‐1 and SW480 cells following TROP2 silencing (M) and TROP2 overexpression (N). (O) Western blotting detection of H3K9la, H3K18la, H4K5la, H4K8la, H4K12la levels in DLD‐1 and SW480 cells following TROP2 overexpression. (P) Western blotting detection of TROP2 and H3K18la in primary tumours from CRC patients without metastasis (P) and matched primary tumours and liver metastases (P and M) from CRLM patients (*n* = 3).

We observed heterogeneous TROP2 upregulation across CRC cell lines versus normal epithelium cells. DLD‐1 and SW480 cells showed high TROP2 expression, while Lovo cells lacked TROP2 (Figure S2A). Unlike receptors with defined ligands, TROP2 signalling is primarily driven by its expression level. Using stable TROP2‐knockdown (Figure S2B) and ‐overexpression (OE; Figure S2C) derivatives of DLD‐1 and SW480, we found that TROP2 depletion significantly inhibited, whereas OE promoted their metastasis‐related capacity, including proliferation (Figure S2D and E), tumourigenicity (Figure S2F and G), and motility (Figure S2H–M). TROP2 silencing restrained in vivo xenograft growth (Figure S2N–P). Analysis of a TROP2‐stratified CRC tissue microarray (*n* = 30; Figure 2A) revealed significant enrichment of glycolysis activation, hypoxia and neovascularisation gene signature in TROP2‐high (H) versus TROP2‐low (L) groups (Figures 2B and S3A). To investigate the in vivo effect of TROP2 expression, we established TROP2 positive (+) and negative (‐) xenograft models using control and TROP2‐OE Lovo cells in immunodeficient mice and then re‐isolated CRC cells for UPLC/MS analysis (Figures 2C and D and S3B and C). Glycolytic intermediates were markedly upregulated in in vivo CRC cells in response to TROP2 expression and its signalling (Figure 2E). A similar pattern was observed in adherent CRC cells cultured in vitro (Figure S3D). TROP2 depletion suppressed glycolytic enzymes, including lactate dehydrogenase A (LDHA) (Figure 2F), and significantly reduced intracellular lactate levels in CRC (Figure 2G), establishing lactate as a critical metabolic effector of TROP2 signalling. TROP2 overexpression promoted excess lactate production (Figure 2H). Concurrently, the extracellular acidification rate (ECAR) was markedly reduced in CRC cells upon TROP2 depletion, with oxygen consumption rate (OCR) being increased (Figure 2I–L). The comparison of the aforementioned metabolomes in SW480 and Lovo cells revealed higher levels of glycolytic intermediates in SW480 (Figure S3E). A much higher intracellular lactate level was observed both in DLD‐1 and SW480 (TROP2‐H) compared to Lovo (TROP2‐L) cell lines, further strengthening our results (Figure S3F). We next investigated whether lactate upregulation in CRC cells triggered histone lactylation (Figure 2M and N). Interestingly, TROP2 depletion decreased while OE significantly upregulated the lactylation levels in CRC cells. We assessed the lactylation status at key histone residues (H4K12la, H4K5la, H3K18la, H4K8la, H3K9la) and demonstrated that histone H3 lysine 18 lactylation (H3K18la) markedly increased in response to TROP2 expression levels (Figure 2O). We examined the H3K18la levels in CRC patient liver metastatic surgical specimens and their match primary lesions. We observed that compared to CRC cases without metastasis, H3K18la was markedly increased in CRLM samples especially liver metastases, in response to their elevated TROP2 expression level (Figure 2P), revealing H3K18la as a biological effector of TROP2 and its signal in CRLM. We also observed a consistent elevation of TROP2 and H3K18la in CRC peritoneal and lung metastases (Figure S3G). These findings collectively revealed that TROP2‐mediated CRC metastasis is featured by H3K18la and might be implicated in metastatic progression.

### 3.3 TROP2 promotes lactate production in CRC through YBX1‐HIF‐1α signalling

**FIGURE 3:**
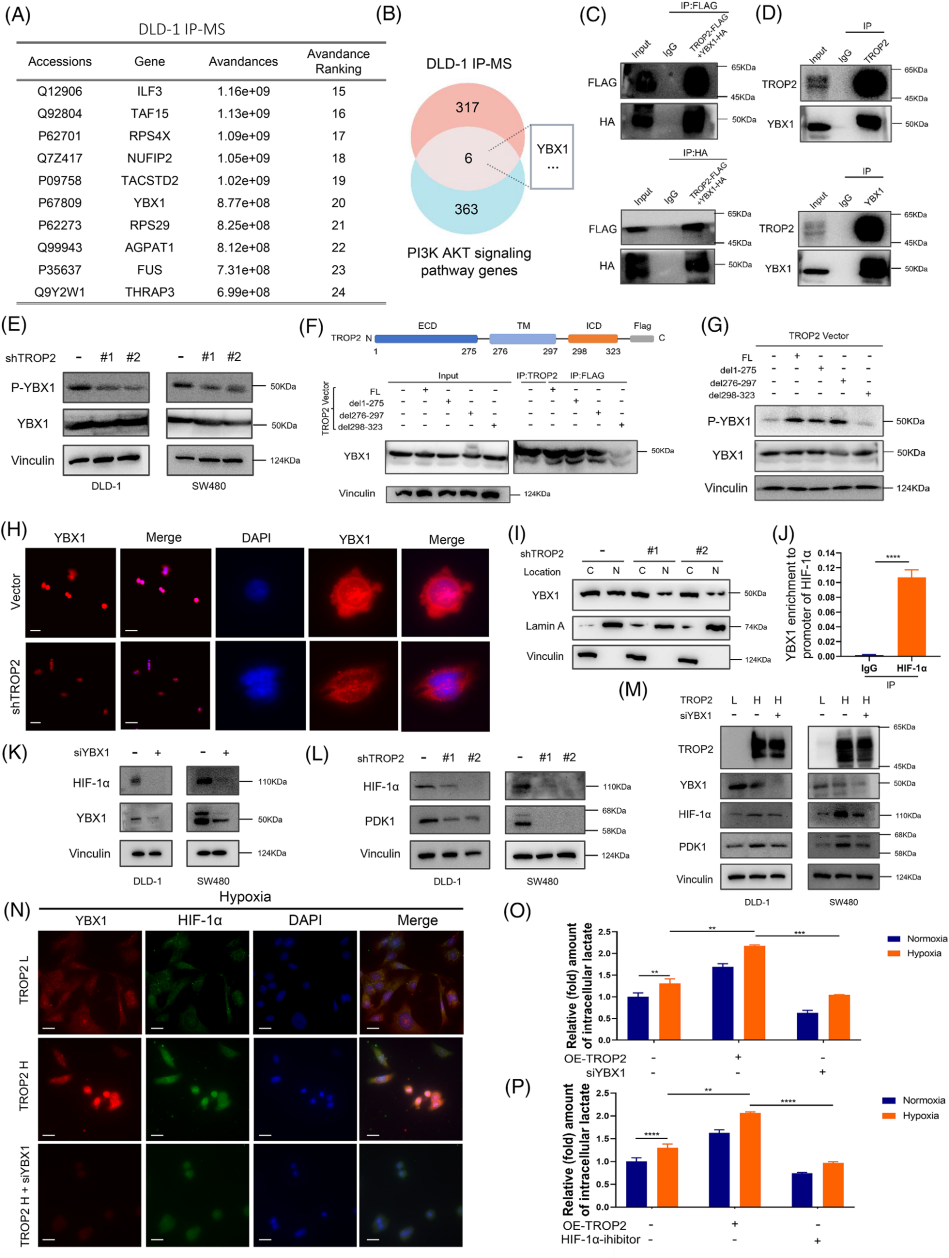
TROP2 promotes lactate production in CRC through YBX1‐HIF‐1α signalling. (**A**) Table of potential TROP2‐interacting proteins identified by IP‐MS in the intracellular environment of CRC DLD‐1 cells (listed in the order of abundance). (B) Strategy to shortlist potential downstream through which TROP2 promotes lactate production. (C) Exogenous interaction between TROP2 and YBX1 was assessed by co‐IP using anti‐FLAG or anti‐HA antibodies in HEK293T cells co‐transfected with TROP2‐FLAG and YBX1‐HA. (D) Endogenous interaction between TROP2 and YBX1 was assessed by co‐IP using anti‐TROP2 or anti‐YBX1 antibodies in SW480 cells. (E) Western blotting detection of P‐YBX1 (ser102) and YBX1 in DLD‐1 and SW480 cells following TROP2 silencing. (F) (Top) Schematic diagram showing the structure and sequence of TROP2‐Flag; (Bottom) SW480 cells were transfected with empty vector, TROP2^FL^‐Flag, TROP2^del1‐275^‐Flag, TROP2^del276‐297^‐Flag and TROP2^del298‐323^‐Flag, respectively. Western blotting analysis was used to detect YBX1 enriched by anti‐TROP2 antibodies (No.1) and anti‐Flag antibodies (No.2‐5) IP. (G) Western blotting detection of p‐YBX1 (ser102) and YBX1 in SW480 cells transfected with empty vector, TROP2^FL^‐Flag, TROP2^del1‐275^‐Flag, TROP2^del276‐297^‐Flag and TROP2^del298‐323^‐Flag, respectively. (H) Images showing changes in the nuclear‐cytoplasmic distribution of YBX1 (red) in SW480 cells following TROP2 silencing. The nucleus is labelled by DAPI (blue) (scale bar: 20 µm). (I) Western blotting detection of the nuclear‐cytoplasmic levels of YBX1 in SW480 cells following TROP2 silencing; C: cytosol, N: nuclear. (J) Immunoprecipitation of DNA fragments (ChIP) from SW480 cells using an YBX1 specific antibody, followed by qPCR analysis with indicated primers; ****p ≤.0001. (K) Western blotting detection of HIF‐1α in DLD‐1 and SW480 cells transfected with siYBX1. (L) Western blotting detection of HIF‐1α and PDK1 in DLD‐1 and SW480 cells following TROP2 silencing. (M) Western blotting detection of HIF‐1α and PDK1 in DLD‐1 and SW480 cells with Low TROP2, High TROP2 and High TROP2+siYBX1. (N) Immunofluorescence staining showing that TROP2 overexpression enhanced while siYBX1 reduced the protein levels of HIF‐1α (green) in hypoxia; (scale bar: 20 µm). (O, P) Abundance of intracellular lactate in TROP2‐OE SW480 cells following siYBX1 (O) or HIF‐1α inhibitor treatment (P) cultured under normoxia or hypoxia for 24 h as measured by a lactate colorimetric kit, normalised to abundance in normoxic group with normal TROP2 expression. Statistical significance was determined using two‐way ANOVA followed by Sidak's multiple comparisons test; **p ≤.01, ***p ≤.001, ****p ≤.0001.

Akt is a required mediator of Trop‐2 downstream signalling.^28^ To determine how TROP2 signalling promoted lactate production, we performed immunoprecipitation combined with mass spectrometry (IP/MS) using TROP2 antibodies on both CRC cells and HEK293T cells (Figures 3A and S4A). Gene ontology (GO) analysis of IP/MS results in CRC cells revealed enrichment in nucleic acid binding, nucleoplasm, protein target pathway (Figure S4B). By overlapping TROP2 IP/MS results from CRC cells and key PI3K‐AKT signalling pathway genes, 6 potential TROP2‐interacting downstreams were identified (Figure 3B), among which YBX1 possessed the highest abundance. YBX1 was also found in the IP/MS results from HEK293T cells, although with significantly lower abundance than in cancer cells (Figure S4A). YBX1 has been identified as a critical glycolytic regulator.^44^, ^45^ We hypothesised that TROP2 promoted lactate production in CRC cells through YBX1 signalling. We substantiated the binding affinity between TROP2 and YBX1 by Co‐IP assay using exogenous proteins in HEK293T cells transfected with TROP2‐FLAG and YBX1‐HA or endogenous proteins in CRC cells (Figure 3C and D). Given that YBX1 is an RNA‐binding protein, we next examined whether RNA mediates its association with TROP2; however, RNase did not appreciably reduce the TROP2–YBX1 interaction (Figure S4C). TROP2 silencing significantly suppressed YBX1 phosphorylation, however did not affect YBX1 expression (Figure 3E). TROP2 expression markedly enhanced the binding of AKT and YBX1 (Figure S4D). Among a group of CRC cell lines with different TROP2 levels but comparable YBX1 baseline, YBX1 phosphorylation level was found higher in TROP2‐high cells (DLD‐1 and SW480) (Figure S4E), supporting our previous findings. We further demonstrated that the TROP2 intracellular domain (ICD; 298–323 aa), but not its extracellular (ECD; 276–297 aa) or transmembrane (TM; 1–275 aa) domains, was necessary for YBX1 binding and its phosphorylation (Figure 3F and G). Phosphorylation of YBX1 at ser102 is a key mechanism driving its nuclear translocation and activation.^46^ We observed that TROP2 depletion markedly inhibited YBX1 nuclear translocation (Figures 3H and I and S4F), indicating YBX1 as a vital TROP2 signal downstream. Nuclear YBX1 acts as a transcription factor, directly activating HIF‐1α transcription in pathological pulmonary hypertension.^47^ We investigated whether TROP2 expression in CRC cells activates YBX1‐HIF‐1α signalling. ChIP‐qPCR revealed marked enrichment of YBX1 at the HIF‐1α promoter (–460 bp to –421 bp) in TROP2‐expressing CRC cells (Figure 3J). TROP2 overexpression in CRC cells boosted this enrichment, which was reversed by the TROP2 (del ICD) mutant (Figure S4G). YBX1 silencing reduced HIF‐1α protein levels (Figure 3K). As expected, TROP2 knockdown significantly reduced both HIF‐1α and its downstream target PDK1 even under normoxia (Figure 3L). Conversely, TROP2 upregulation enhanced HIF‐1α and PDK1 expression; this effect was reversed by YBX1 knockdown (Figure 3M). Hypoxic immunofluorescence showed that TROP2 further promoted HIF‐1α expression and activation in a YBX1‐dependent manner under hypoxia (Figures 3N and S4H). Critically, both siYBX1 and HIF‐1α inhibition blocked the TROP2 overexpression‐induced increase in lactate levels under normoxia and hypoxia (Figure 3O and P). In stable YBX1‐ and HIF‐1α‐knockdown CRC cells (Figure S4I), TROP2 overexpression failed to significantly upregulate lactate production (Figure S4J) and H3K18la levels (Figure S4K), further supporting the role of YBX1/HIF‐1α in TROP2‐mediated lactate production. These data demonstrate that TROP2 expression in CRC cells modulates the YBX1‐HIF‐1α signalling axis to promote lactate production.

### 3.4 Inhibition of H3K18la inhibits TROP2‐mediated CRC liver metastatic progression

**FIGURE 4:**
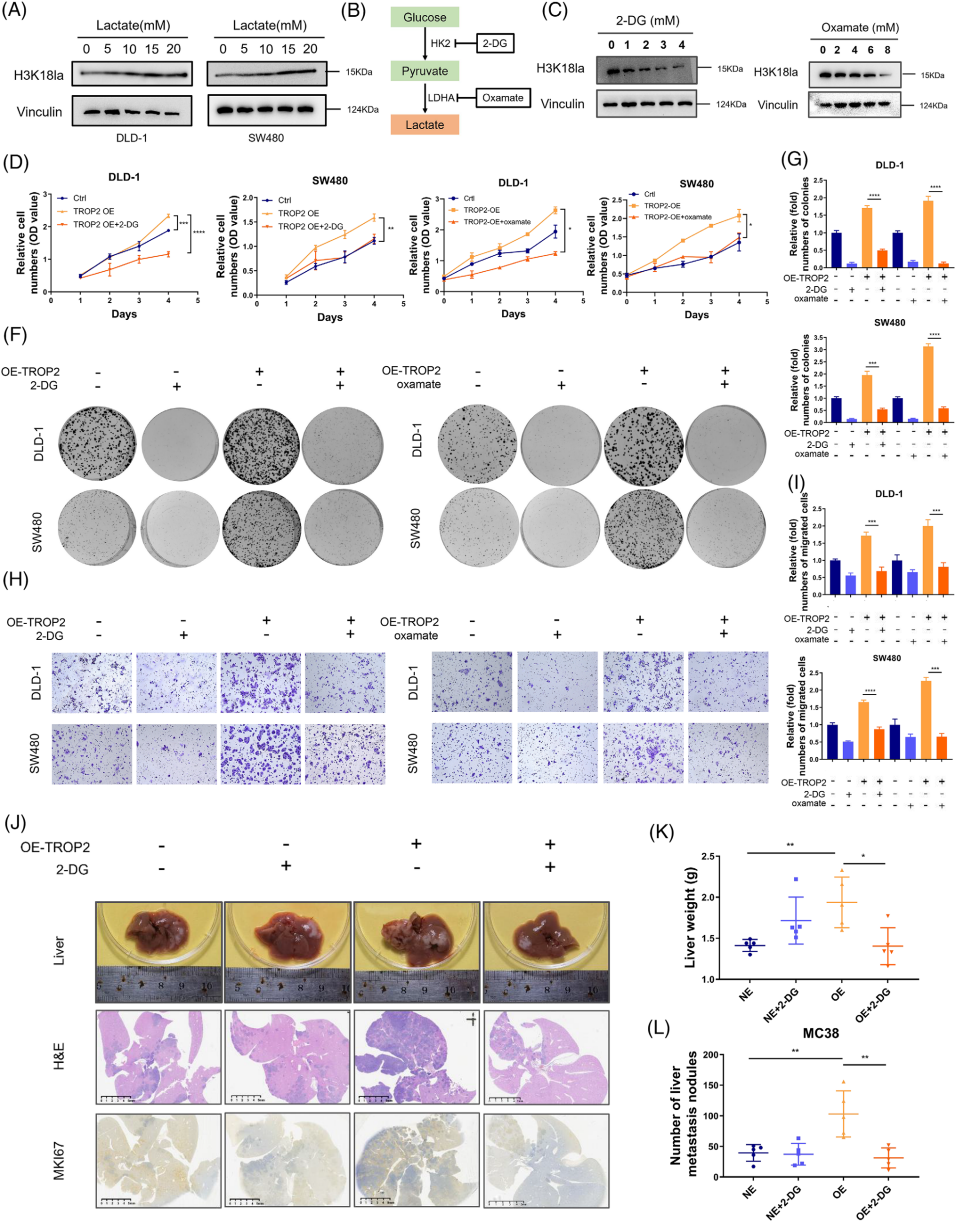
Inhibition of H3K18la inhibits TROP2‐mediated CRC liver metastatic progression. (A) Western blotting detection of H3K18la in DLD‐1 and SW480 cells cultured in gradient concentrations of lactate for 24 h. (B) Strategies to inhibit lactate production and H3K18la. (C) Western blotting detection of H3K18la in SW480 cells cultured in gradient concentrations of 2‐DG or oxamate for 24 h. (D, E) Proliferation changes in TROP2‐overexpressing DLD‐1 and SW480 cells cultured in 2 mM 2‐DG (D) or 4 mM oxamate (E) were evaluated by CCK8 assay; *p ≤.05, **p ≤.01, ***p ≤.001; OE, overexpression. (F) Tumourgenicity changes of TROP2‐OE DLD‐1 and SW480 cells cultured in 2 mM 2‐DG or 4 mM oxamate were evaluated by colony formation assay and compared to cells with normal TROP2 expression. (G) Statistic analysis of colony formation assay in TROP2‐OE DLD‐1 and SW480 cells cultured in 2‐DG or oxamate; ***p ≤.001,****p ≤.0001. (H) Cell migration changes of TROP2‐OE DLD‐1 and SW480 cells cultured in 2 mM 2‐DG or 4 mM oxamate, migrating from serum‐free to 20% FBS chambers, compared to cells with normal TROP2 expression. (I) Quantification of migrated cells; ***p ≤.001,****p ≤.0001. (J) Representative images of liver metastasis changes in TROP2‐OE MC38 cells after splenic injection under 2‐DG treatment compared to cells with normal TROP2 expression, showing gross liver, H&E staining, and MKI67 IHC staining of each group (scale bar: 5 mm). (K) Liver weights in the specified groups (*n* = 5 each group); *p ≤.05, **p ≤.01. (L) Numbers of liver metastasis nodules in the specified groups; **p ≤.01.

Gradient lactate treatment elevates H3K18la levels in CRC cells (Figure 4A). We next suppressed intracellular lactate production using glycolytic inhibitors (2‐deoxy‐D‐glucose [2‐DG] /oxamate) to investigate whether block of histone lactylation in CRC cells could inhibit their metastatic progression driven by TROP2 (Figure 4B). Notably, 2‐DG or oxamate treatment caused a dose‐dependent decline in cellular lactate production and H3K18la levels across CRC cells (Figures 4C and S5A and B). H3K18 lactylation depletion in vitro markedly suppressed TROP2‐driven pro‐metastatic capacities in CRC cells, including proliferation (Figure 4D and E), tumourigenicity (Figure 4F and G) and motility (Figure 4H and I). Silencing MCT4, a lactate transporter, inhibited H3K18la and motility in TROP2‐overexpressing CRC cells consistent with glycolytic inhibitors (Figures S5B and C and S5E and F). Conversely, in TROP2‐knockdown CRC cells, the addition of exogenous lactate restored both H3K18la levels and their metastatic capacity (Figures S5D and S5G and H), supporting the role of lactate and H3K18la in CRC metastatic ability. Metastatic colonisation is a critical rate‐limiting step in metastatic progression,^48^ with hypoxia involved in the liver microenvironment which metastatic cells must overcome.^49^ We then exposed CRC cells to hypoxia and then characterised the lactate changes. TROP2‐H CRC cells exhibited accelerated lactate production compared to TROP2‐L cells under sustain hypoxia, which peak at 24 h (Figure S5I and J). H3K18la inhibition also significantly suppressed pro‐metastatic phenotypes driven by TROP2 in CRC cells under hypoxia (Figure S5K–O). To characterise the role of H3K18la in the TROP2‐mediated liver metastatic progression in vivo, we performed intra‐splenic injection‐based liver metastatic colonisation assays. Equal amounts of TROP2‐L and TROP2‐H MC38 cells were splenic injected into C57BL/6 mice with or without H3K18la inhibition using 2‐DG treatment. As expected, TROP2 overexpression enhanced liver metastatic progression potential, evidenced by increased average liver weight and metastasis nodules. Notably, H3K18la inhibition with 2‐DG reversed these effects in TROP2‐high groups (Figures 4J and S5P), significantly reducing both average liver weight (Figure 4K) and the number of metastasis nodules (Figure 4L). While only a mild effect was observed on TROP2‐low groups, H3K18la inhibition effectively abolished the TROP2‐driven enhancement of liver metastasis. Concurrently, H3K18la inhibition markedly suppressed proliferation in TROP2‐high‐driven CRLM lesions, as assessed by Ki‐67 immunohistochemistry (Figure 4J). Together, these data indicated that histone lactylation plays a role in TROP2‐driven metastatic progression and inhibition of H3K18 lactylation efficiently inhibits TROP2‐mediated liver metastatic colonisation in experimental models.

### 3.5 Genome‐wide analysis of the transcriptional consequences of H3K18la in TROP2‐high colorectal cancer

**FIGURE 5:**
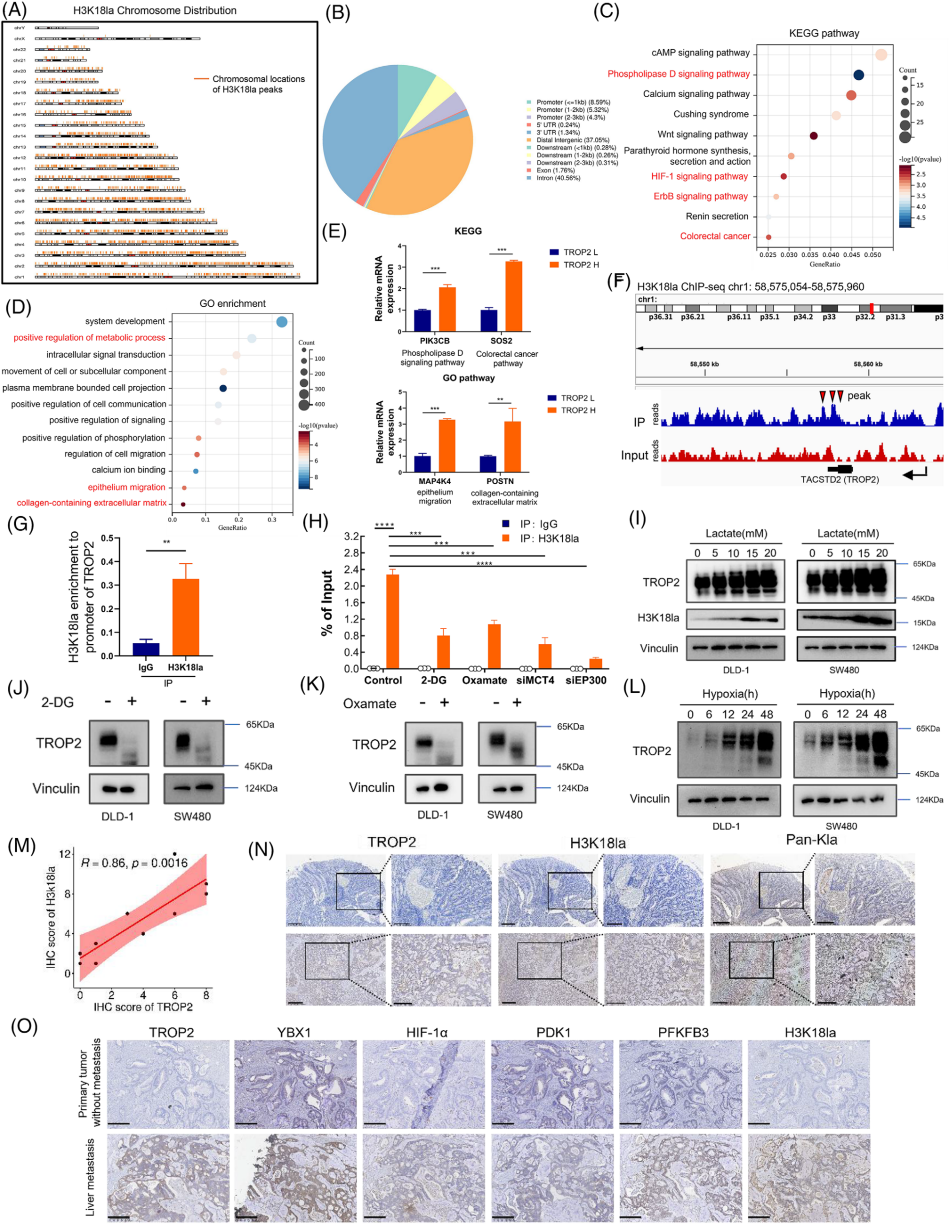
Genome‐wide analysis of the transcriptional consequences of H3K18la in TROP2‐high colorectal cancer. (A) Chromosome distribution plot showing chromosomal locations of H3K18la peaks in TROP2‐high CRC cells identified by H3K18la ChIP‐seq. (B) Genome‐wide distribution of H3K18la‐binding peaks in TROP2‐high CRC cells. (C) KEGG pathway analysis of H3K18la peaks in TROP2‐high CRC cells. (D) GO enrichment analysis of H3K18la peaks in TROP2‐high CRC cells. (E) Expression of representative pro‐metastatic KEGG pathway genes (PIK3CB, SOS2) and GO pathway genes (MAP4K4, POSTN) were detected in CRC cells with low or high TROP2 expression by RT‐qPCR; **p ≤.01, ***p ≤.001. (F) IGV tracks displaying enriched H3K18la modifications at the TROP2 promoter region by ChIP‐seq Analysis. (G) Immunoprecipitation of DNA fragments (ChIP) from SW480 cells using H3K18la specific antibody, followed by qPCR analysis with indicated primers; **p ≤.01. (H) ChIP‐qPCR analysis for H3K18la status at the TROP2 promoter of SW480 cells treated with 4 mM 2‐DG, 8 mM oxamate or transiently transfected with siRNAs targeting MCT4 or EP300 for 24 h. ***p ≤.001, ****p ≤.0001. (I) Western blotting detection of TROP2 in DLD‐1 and SW480 cells cultured in gradient concentrations of lactate for 24 h. (J, K) Western blotting detection of TROP2 in DLD‐1 and SW480 cells cultured in 2 mM 2‐DG (J) and 4 mM oxamate (K) for 24 h. (L) Western blotting detection of TROP2 in DLD‐1 and SW480 cells exposed to hypoxia (1% oxygen) at indicated time points before harvest. (M) Correlation of TROP2 expression with H3K18la levels in CRC tissues, assessed by person's correlation analysis. (N) Representative cases demonstrating the correlation of TROP2, Pan‐Kla and H3K18la levels in CRC tissues, analysed by immunohistochemistry (IHC) staining.(scale bar: black, 400 µm; red, 200 µm). (O) Representative cases demonstrating the activation of TROP2 /H3K18la pathway loop in CRC liver metastasis compared to primary tumour without metastasis, analysed by immunohistochemistry (IHC) staining (scale bar: 200 µm).

Histone lactylation plays a vital role in regulating gene transcription. We next performed a genome‐wide H3K18la chromatin immunoprecipitation sequencing (ChIP‐seq) approach to identify genes that are potentially regulated by this histone mark in TROP2‐high CRC. A total of 4251 marked H3K18la peaks were identified using ChIP‐seq detection (Figure 5A). Among these peaks, 8.59% resided within 1 kb of transcription start sites (TSSs), and a further 5.32% and 4.3% were located 1–2 kb and 2–3 kb upstream, respectively (Figure 5B). The H3K18la peaks were significantly enriched in multiple Kyoto Encyclopedia of Genes and Genomes (KEGG) pathways associated with metastasis and survival, including phospholipase D signalling, HIF‐1 signalling, ErbB signalling and colorectal cancer pathways (Figure 5C). GO analysis revealed enrichment in biological processes linked to positive regulation of metabolism, epithelial cell migration, and collagen‐containing extracellular matrix signalling, processes implicated in enhancing liver metastatic colonisation and progression of CRC cells (Figure 5D). Given that the levels of H3K18la were elevated in TROP2 high CRC cells and hyperlactylated H3K18la was enriched in genes related to survival and liver metastatic progression, we next determined whether these genes were increased in response to TROP2 expression. Representatively, ChIP‐seq showed that H3K18la was enriched at the promoter of a set of critical pro‐survival and pro‐metastatic genes including PIK3CB, SOS2, MAP4K4, POSTN, which are involved in the aforementioned pathways. qPCR assays confirmed their marked increase in TROP2‐high CRC cells (Figure 5E). Together, these data indicated that H3K18la serves as a molecular switch promoting multiple pro‐metastatic and pro‐survival genes transcription in CRC cells in response to TROP2 and participates in CRC metastatic progression.

### 3.6 Lactate sustains TROP2 expression in CRC cells via H3K18la

TROP2 is expressed during embryonic development but substantially downregulated in normal adult tissues, with epigenetic mechanisms involved in its regulation.^50^ Interestingly, we noted that TROP2 possessed an enrichment of H3K18la peak in the promoter position within CRC cells (Figure 5F). ChIP‐qPCR further confirmed H3K18la's enrichment at TROP2 promoter (Figure 5G), which was reduced by glycolytic inhibitor, siRNA for lactate transporter MCT4 or histone lactylation writer EP300 (Figure 5H), indicating that H3K18la might participate in sustaining TROP2 expression in CRC cells, thereby forming a positive feedback loop. To test this, we modulated lactate and H3K18la levels by treating cells with lactate or glycolytic inhibitors (2‐DG or oxamate) and assessed changes in TROP2 expression. Increasing lactate concentrations enhanced TROP2 levels, whereas 2‐DG or oxamate treatment suppressed it (Figure 5I–K). Moreover, siRNA for MCT4 or EP300 consistently inhibited TROP2 expression (Figure S6A and B). Notably, under sustained hypoxia, CRC cells exhibited a time‐dependent accumulation of TROP2 protein compared to normoxic controls (Figure 5L). We further revealed a significant positive correlation between TROP2 and H3K18la levels in primary tumours or matched liver metastases (Figure 5M and N). Moreover, immunohistochemical (IHC) analysis of paraffin‐embedded clinical specimens demonstrated markedly elevated activation of the TROP2/YBX1/HIF‐1α/PDK1/PFKFB3/H3K18la axis in liver metastatic lesions relative to primary tumours without metastasis, among which PDK1 and PFKFB3 represent the key glycolytic downstream of HIF‐1α (Figure 5O). Collectively, these data established that lactate mediates TROP2 expression in CRC cells via H3K18la, and supported the existence of a TROP2/YBX1/HIF‐1α/H3K18la positive feedback loop which promotes CRC metastatic progression.

### 3.7 Acriflavine suppresses TROP2‐driven CRLM progression by targeting the TROP2/YBX1/HIF‐1α/H3K18la feedback loop

**FIGURE 6:**
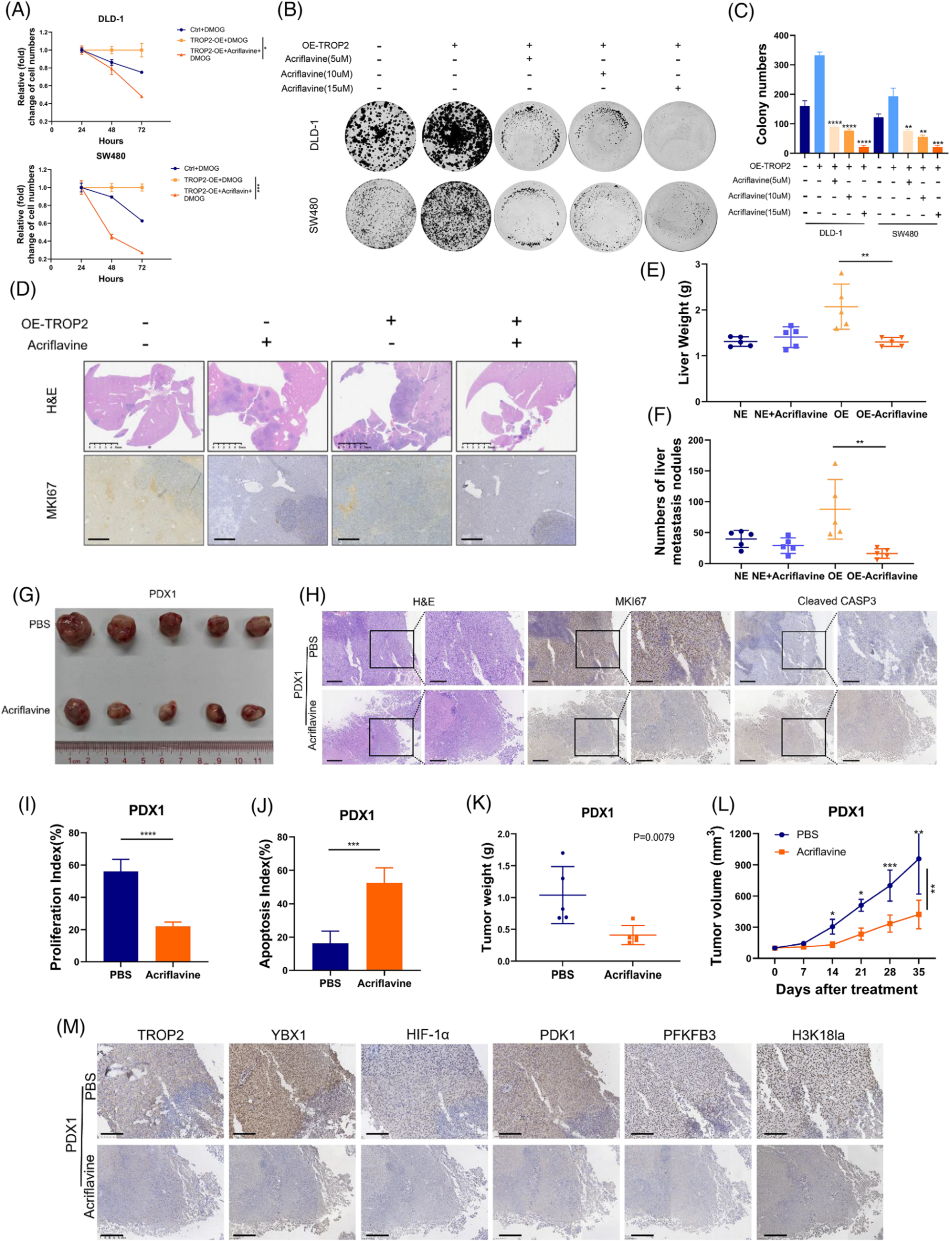
Acriflavine suppresses TROP2‐driven CRLM progression by targeting the TROP2/YBX1/HIF‐1α/H3K18la feedback loop. (A) Proliferation changes in TROP2‐overexpressing DLD‐1 and SW480 cells treated with 2 mM DMOG ± 10 µM acriflavine were evaluated by CCK8 assay and compared to cells with normal TROP2 expression; OE, overexpression; *p ≤.05, ***p ≤.001. (B) Tumourgenicity changes of TROP2‐OE DLD‐1, SW480 cells treated with gradient concentrations of acriflavine were evaluated by colony formation assay and compared to cells with normal TROP2 expression. (C) Statistic analysis of colony formation assay in TROP2‐OE DLD‐1, SW480 cells treated with gradient concentrations of acriflavine. All of the experiments were performed in triplicate, and relative colony numbers are shown as means ± SD; ***p ≤.001. (D) Representative images of liver metastasis changes in TROP2‐OE MC38 cells after splenic injection under acriflavine treatment compared to cells with normal TROP2 expression, showing H&E staining, and representative MKI67 IHC staining of each group (scale bar: Top, 5 mm; Bottom, 200 µm). (E) Liver weights in the specified groups (*n* = 5 each group); **p ≤.01. (F) Numbers of liver metastasis nodules in the specified groups; **p ≤.01. (G) Photographic comparison of patient‐derived xenograft #1 (PDX1) tumours with PBS or acriflavine treatment. (H) Representative images of H&E and IHC staining for MKI67 or cleaved CASP3 in PDX1 tumours (scale bar: left, 400 µm; right, 200 µm). (I, J) Quantification of proliferation index (I) and apoptosis index (J) between groups for PDX1. (K) PDX1 tumour weights between groups. (L) Growth curves of PDX1 tumours between groups. (M) Representative paraffin‐embedded sections of PDX1 tumours for each group stained with antibodies against TROP2, YBX1, HIF‐1α, PDK1, PFKFB3, H3K18la (scale bar: 200 µm).

Acriflavine, a FDA‐approved inhibitor of HIF‐1α, demonstrated promising therapeutic effects in controlling the progression of various diseases, including cancer.^51^, ^52^ Acriflavine treatment substantially suppressed TROP2‐mediated lactate production in CRC cells (Figure 3P). Herein, we investigated whether acriflavine restrains TROP2‐driven CRLM progression through inhibition of lactate and disruption of the TROP2/YBX1/HIF‐1α/H3K18la feedback loop. We found that acriflavine treatment of multiple CRC cell lines effectively inhibited TROP2‐driven proliferation (Figure 6A), tumourigenicity (Figures 6B and C and S7C and D) and migration (Figure S7A and B). In the MC38 intra‐splenic injection‐based liver metastatic model, acriflavine treatment markedly inhibited TROP2‐driven CRC liver metastatic colonisation, as evidenced by fewer liver metastasis nodules (Figures 6D and F and S7E), reduced liver weight (Figure 6E), and lower MKi67 expression levels (Figure 6D). To evaluate the preclinical efficacy of acriflavine against TROP2‐driven colorectal liver metastases, we established two patient‐derived xenograft (PDX) models using specimens from two patients with TROP2‐high CRLM (Figures 6G and S7F). Acriflavine‐mediated HIF‐1α inhibition significantly suppressed tumour proliferation and concurrently increased apoptosis in both PDX tumours, as evidenced by Ki‐67 and cleaved caspase‐3 staining (Figures 6H–J and S7G–I). Acriflavine treatment effectively suppressed PDX tumours growth, evidenced by markedly decreased tumour weight (Figures 6K and S7J) and volume (Figures 6L and S7K). Furthermore, acriflavine effectively inhibited the TROP2/YBX1/HIF‐1α/H3K18la signalling axis in both PDXs (Figures 6M and S7L). Knockdown of HIF‐1α in TROP2‐high CRC cells phenocopied acriflavine in inhibiting their migration (Figure S8A and B). Both shRNAs targeting HIF‐1α inhibited the entire axis in TROP2‐high CRC cells (Figure S8C). Beyond tumour‐intrinsic mechanisms, we further examined the tumour immune microenvironment's (TIME) contribution to acriflavine's anti‐metastatic effect in the above MC38 model. No significant differences in CD3, CD8, Foxp3, or CD206 abundance were observed between TROP2‐high acriflavine and control groups (Figure S8D and E), indicating a minor role for TIME. These data collectively demonstrate that acriflavine suppresses TROP2‐high CRC liver metastatic colonisation and progression by disrupting the TROP2/YBX1/HIF‐1α/H3K18la signalling axis, suggesting its potential as a promising therapeutic strategy for TROP2‐high colorectal cancer.

## 4 DISCUSSION

Histone lactylation, a newly recognised epigenetic mark driven by lactate,^29^ represents a novel mechanism that converts cellular metabolic signatures into transcriptional landscapes through modulating gene transcription. Lactylation has been found orchestrating diverse cellular physiological and pathological processes including cancer development, diabetes, infection, and Alzheimer's disease. In this study, we discovered a positive feedback loop between H3K18la and TROP2 signal existing within CRC metastases that promoted CRC liver metastatic colonisation and progression. Elevated TROP2 in liver metastases was identified highly linked to early post‐hepatectomy recurrence and poor prognosis in clinical cohorts. Mechanistically, elevated TROP2 expression in CRC promoted excess lactate production via YBX1/HIF‐1α signalling and stimulated histone lactylation at H3 lysine 18 (H3K18la), which promoted multiple pro‐metastatic genes transcription. Inhibition of H3K18la markedly suppressed TROP2‐mediated CRLM progression. Furthermore, TROP2‐induced H3K18la in turn sustained TROP2 expression, forming a feedback loop and further promoting the progressive behaviours of CRC within the metastatic microenvironment. Interruption of this pathway by targeting HIF‐1α signalling efficiently suppressed TROP2‐driven CRLM progression, as confirmed by multiple pre‐clinical models. These findings might suggest a novel strategy to improve TROP2‐high CRC metastasis treatment and prevent metastatic recurrence by disrupting this feedback loop.

Distant metastasis and post‐treatment recurrence represent the most lethal aspects of CRC, with liver metastasis being the predominant pattern. While curative resection combined with chemotherapy constitutes standard care for CRLM,^53^ only a subset of patients are surgical candidates, achieving 5‐year survival rates of approximately 30%.^54^, ^55^ Non‐surgical candidates face an even poorer prognosis. Although modern therapeutic regimens improve initial response rates, resistance to fluoropyrimidine‐platinum therapies inevitably emerges. Consequently, deeper insights into the molecular drivers of CRC metastasis are essential for developing more precise and durable therapies.^41^ As a known stem cell marker, TROP2 is expressed at low levels in normal mature tissues. During cancer development, its expression can be induced by multiple signalling pathways, including KRAS mutation,^56^ Tumour Necrosis Factor (TNF),^57^ and transforming growth factor (TGF).^58^ Concurrently, epigenetic dysregulation^59^ serves as another critical regulatory layer contributing to its aberrant expression in malignancies. While the contribution of TROP2 to cancer development in solid tumours has been recognised for decades,^11^ key aspects of its biology in metastasis remain poorly defined. Specifically, the expression dynamics of TROP2 during tumour progression and metastasis, its heterogeneity among individual patients, and its associated clinical significance are still unclear. Recent studies have identified high TROP2 levels in advanced non‐small cell lung cancer as a major prognostic factor associated with primary resistance to immune checkpoint blockade (ICB), but not with chemotherapy sensitivity.^16^ Elevated TROP2 expression in CRC has also been linked to refractory disease, microsatellite stable (MSS) status, and tumour microenvironment remodelling.^13^, ^17^ Notably, a pioneering study identifying cell subpopulations mediating postoperative recurrence in a human‐like mouse model found TROP2 to be the most markedly reactivated fetal intestine‐like progenitor signature in liver recurrent cells.^19^ Our findings demonstrated that TROP2 expression was upregulated during CRC metastasis development and served as a critical prognostic indicator in both oligometastatic (initially resectable) and non‐oligometastatic CRLM cases. As reported, TROP2 functions as a cell surface receptor incorporated in multiple oncogenic pathways,^11^ especially AKT signal.^28^ However, it may simultaneously serve as an oncogene^28^ and tumour suppressor gene^50^ in various cancers in a context‐dependent manner. In our study, we for the first time established lactate as a key metabolic effector of TROP2 signalling and identified H3K18la as a TROP2 signalling‐responsive modification mark using both in vitro and in vivo approaches. Moreover, we identified YBX1 as a novel TROP2 downstream through which TROP2 directly activated HIF‐1α signal, thereby promoting excess lactate production. Our study delivered a new exploration and important supplement of TROP2 signalling and linked TROP2 signal to metabolic reprogramming‐epigenetic regulation.

Emerging evidence positions cellular metabolism as a central determinant of cell fate, operating beyond its canonical role in sustaining viability to instruct and reprogram cellular behaviour.^60^, ^61^ TROP2 was elevated in CRC metastasis and TROP2 signalling promoted excess lactate production, further enhanced by the liver hypoxic stress; However, the role of lactate accumulated within liver metastatic microenvironment in CRC liver metastatic progression remains unclear. Growing studies highlight the crosstalk between metabolic substrates and histone modifications.^62^, ^63^, ^64^ Similar to other modification, histone lactylation has been proved to directly promote gene expression by altering chromatin structure and facilitating the recruitment of transcriptional machinery. Our data revealed that lactate contributes to TROP2‐driven CRLM colonisation and progression by elevating H3K18la and facilitating multiple pro‐metastatic and pro‐survival gene transcriptional programs. Intra‐splenic injection‐based liver metastatic colonisation assay is an established model assessing liver metastasis. Using this model, we revealed H3K18la reduction suppressed CRLM progression. Genome‐wide ChIP‐seq was used to identify targeted genes regulated by H3K18la in TROP2‐high CRC cells. H3K18la peaks were deposited at promoters of pro‐metastatic genes, and pathway analyses revealed enrichment of proliferation, migration and collagen‐relating pathways – key effectors of metastasis. Interestingly, the enriched pathways involve classical TROP2 functions – system development, Wnt signalling and Ca^2^⁺ signalling. These findings position H3K18la as a metabolically responsive epigenetic switch that enables pro‐metastatic transcription downstream of TROP2 signalling. We next continued to explore the mechanism of how TROP2 was elevated upon CRC metastasis development. TROP2 is regulated by epigenetic mechanisms. In the present study, we observed an enrichment of H3K18la peak in the promoter of TROP2. Given that YBX1 is a 5‐methylcytosine (m5C) mRNA reader,^65^ we further considered the possibility that YBX1 recognises m5C modifications on TROP2 mRNA and modulates its stability and expression. Previous research did not find significant m5c mark in TROP2 mRNA in Hela cells^66^ (TROP2‐H^67^). Our m5C meRIP‐qPCR assay in CRC cells did not find evident m5C enrichment on TROP2 mRNA (Figure S9A) compared to positive control^68^ and modulating YBX1 did not significantly affect TROP2 mRNA stability (Figure S9B), which further strengthens our findings. Collectively, our results showed that TROP2‐induced (dependent on HIF‐1α signal) H3K18la in turn sustains TROP2 expression, thereby establishing a positive feedback loop that promotes CRLM progression.

Acriflavine, an FDA‐approved inhibitor of HIF‐1α, inhibits HIF heterodimerisation and subsequent transcriptional activation, thereby inhibiting HIF‐1α‐mediated lactate production. In recent years, it has been found to have promising therapeutic effects in controlling the progression of various diseases, including cancer.^51^, ^52^, ^69^ In our results, pharmacological inhibition of HIF‐1α activity with acriflavine demonstrated remarkable efficacy in TROP2‐high intra‐splenic injection‐based liver metastatic and PDX pre‐clinical models. Given that TROP2‐high CRC exhibited elevated HIF‐1α‐dependent lactate production activity to support metastatic progression, combining acriflavine with chemotherapy, or other targeted therapies may provide a promising strategy to prevent disease progression, thereby prolonging survival of CRC patients with metastasis.

**FIGURE 7:**
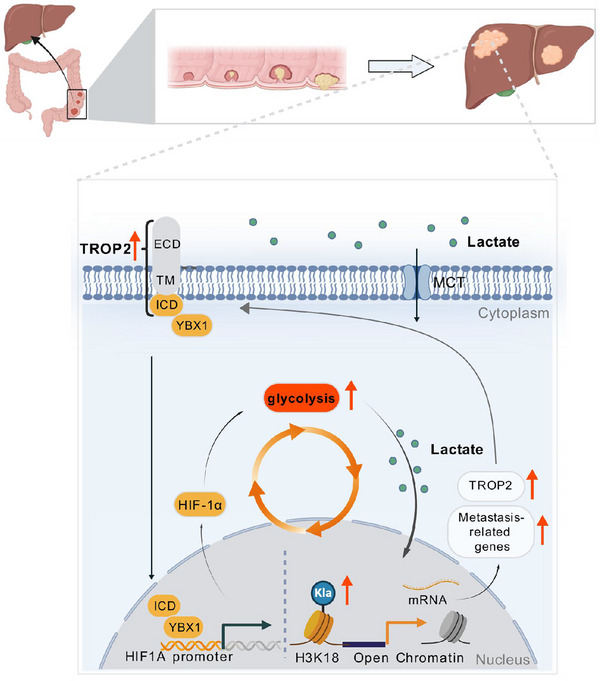
Schematic diagram of how a TROP2/glycolysis/H3K18la feedback loop in colorectal cancer cells modulates liver metastasis. In colorectal cancer (CRC) cells, elevated TROP2 promotes glycolysis and lactate production through YBX1/HIF‐1α signalling. Accumulated lactate within metastatic environment increases histone H3 lysine 18 lactylation that transcriptionally upregulates TROP2 and metastatic‐related genes, thereby forming a positive feedback loop and contributes to CRC liver metastatic progression.

In summary, during the CRLM process, upregulated TROP2 promoted excess lactate production via the YBX1‐HIF‐1α signalling and increased H3K18la as a TROP2‐responsive epigenetic switch activating a set of pro‐metastatic gene transcriptions. TROP2‐induced H3k18la in turn sustained TROP2 expression to form a positive feedback loop, thereby promoting liver metastatic colonisation and progression (Figure 7). Our study suggested that the pharmacological inhibition of HIF‐1α with acriflavine may offer a novel approach to improve the clinical outcomes of TROP2‐high CRC metastasis patients.

## AUTHOR CONTRIBUTIONS

WFW, YXD, WHL, and JHP designed this study. WFW, YXD, and WHL performed most of the molecular and animal experiments, drafted the manuscript, and created the diagrams. RWW, CZ, YBX, JHH, and LY offered assistance with experiment skills. LEL, and JL performed statistical analyses. DK and WLZ discussed the data and assisted with manuscript revision. QJO and ZZP provided clinical and experimental resources. JHP, JZL, PRD, and YJF provided financial and resource support and supervised this study. All authors read and approved the final manuscript.

## CONFLICT OF INTEREST STATEMENT

The authors declare no competing interests.

## ETHICS STATEMENT

Informed consent for the use of specimen, imaging and clinical data was obtained from the patients. The study was approved by the Institutional Research Ethics Committees of Sun Yat‐sen University Cancer Center (Guangzhou, China, approval number: GZR2020‐071).

All mice experimental procedures adhered to the National Institutes of Health Guide for the Care and Use of Laboratory Animals (NIH Publications No. 8023, revised 1978) and were approved by the Animal Care and Use Committee of Sun Yat‐sen University (L102022023001K, Guangzhou, China).

## Supporting information



Supporting Information

Supporting Information

Supporting Information

Supporting Information

Supporting Information

Supporting Information

## Data Availability

All data generated in this study are available upon request from the corresponding author.
