# Supplementary material for: Feedback regulation between histone H3 lysine 18 lactylation and TROP2‐mediated glycolysis drives metastatic progression of colorectal cancer
Source: Clin Transl Med. 2026 Jan 3;16(1):e70562. doi: 10.1002/ctm2.70562 (PMC12761367; doi:10.1002/ctm2.70562)
Supplement: Supplementary file 1 — Supporting Information [file CTM2-16-e70562-s002.docx]

**Supplemental Text and Figures**

**
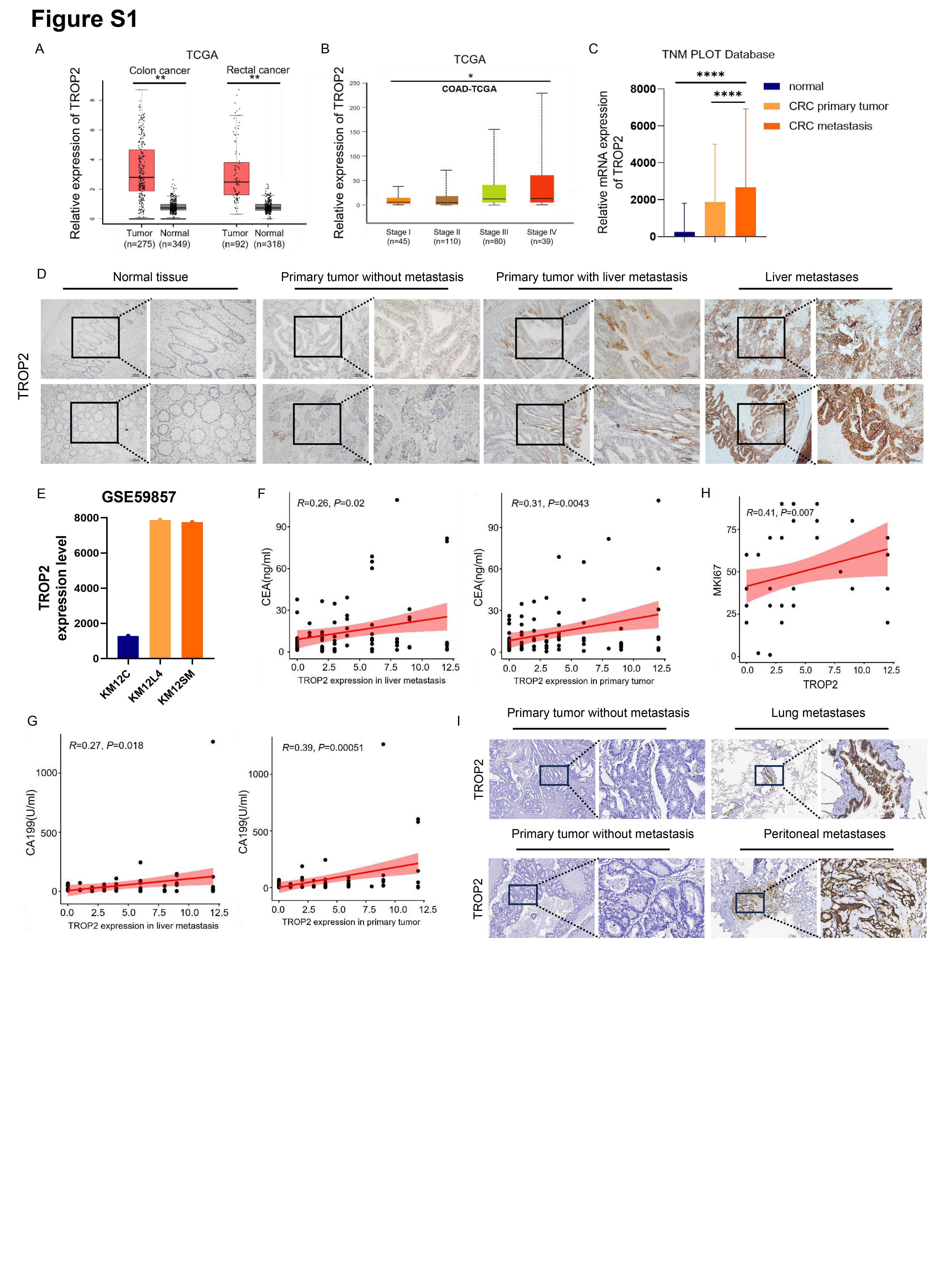
**

**Figure S1.** Elevated TROP2 expression in colorectal cancer liver metastases (CRLM) predicts post-hepatectomy recurrence and poor prognosis. (**A**). Analysis of *TROP2* expression in colorectal cancer (CRC) and adjacent normal tissues from TCGA database, assessed by *t* test. (**B**). *TROP2* expression analysis across CRC stages from TCGA database, assessed by t test. (**C**). Analysis of *TROP2* expression in adjacent normal tissues, CRC primary tumor, metastasis from TNM PLOT Database, assessed by *t* test. (**D**). Representative cases demonstrating high versus low TROP2 expression in matched primary tumors and liver metastases from CRLM patients (n=2), primary tumors from CRC patients without metastasis (n=2), and normal tissues, analyzed by immunohistochemistry (IHC) staining.(scale bar: 100 μm;). (**E**). TROP2 expression comparation of KM12L4, KM12SM cell lines and their parental cell KM12C. (**F**). Correlation of TROP2 expression in hepatic metastasis (left) and primary lesion (right) with serum CEA levels in CRLM cohort 1, assessed by person’s correlation analysis. (**G**). Correlation of TROP2 expression in hepatic metastasis (left) and primary lesion (right) with serum CA199 levels in CRLM cohort 1, assessed by spearman’s (left) and person’s (right) correlation analysis. (**H**). Correlation of TROP2 and MKI67 expression levels in primary tumors of CRLM cohort 1, assessed by person’s correlation analysis. (**I**). Representative cases demonstrating high versus low TROP2 expression in lung and peritoneal metastases and primary tumors from CRC patients without metastasis.

**
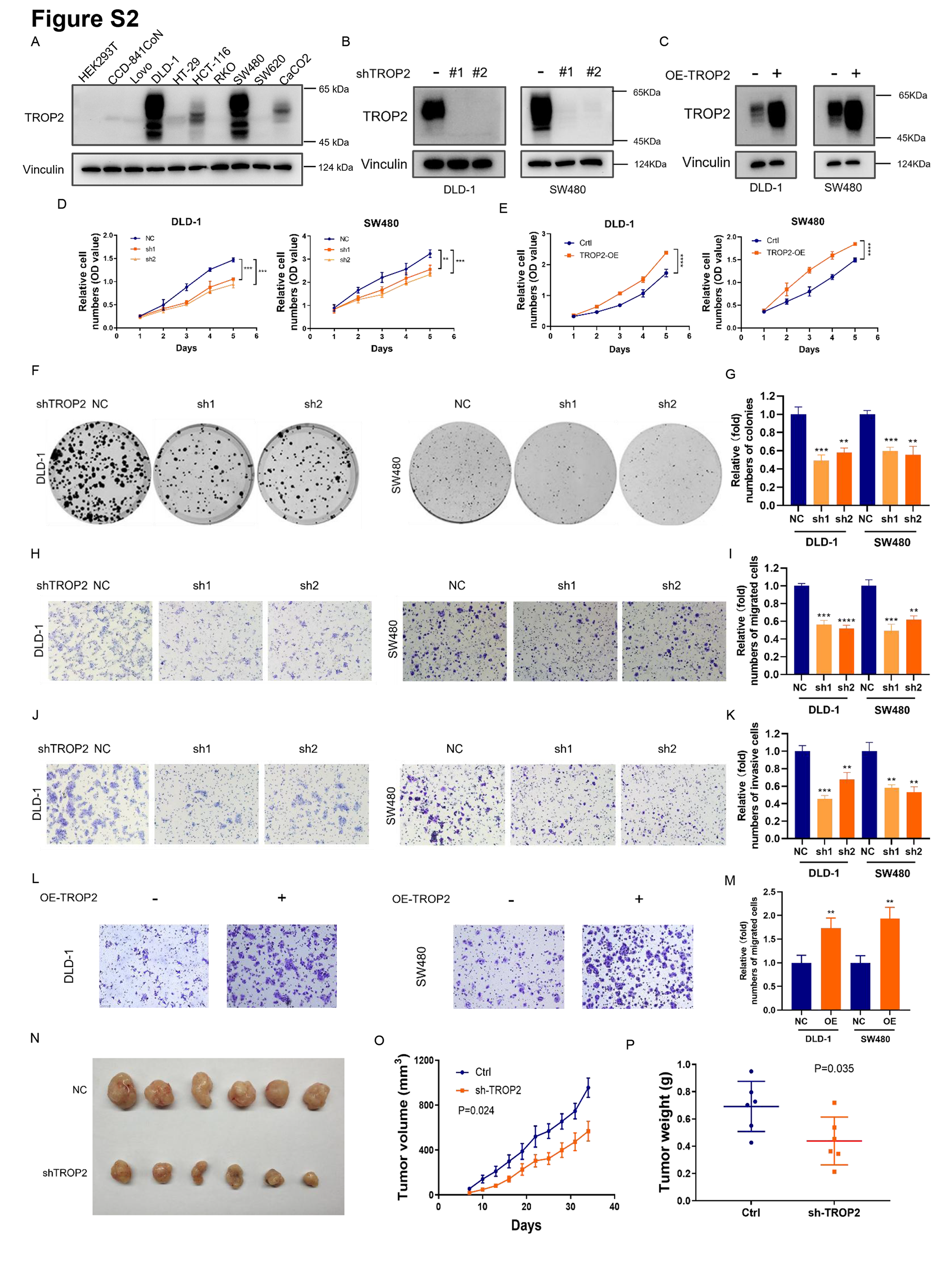
**

**Figure S2.** TROP2 promotes CRC cell malignant phenotypes. (**A**). Western blotting detection of TROP2 in normal colon epithelial cells and CRC cells. (**B**). Western blotting detection of TROP2 in DLD-1 and SW480 cells transfected with shTROP2. (**C**). Western blotting detection of TROP2 in DLD-1 and SW480 cells with or without TROP2 overexpression. (**D** and **E**). Proliferation of DLD-1 and SW480 cells following TROP2 silencing (D) or TROP2 overexpression (E) was assessed via CCK8 assay; OD: Optical Density; ***P* ≤0.01, ****P* ≤0.001, *****P*≤0.0001. (**F**). Tumorigenicity of DLD-1 and SW480 cells following TROP2 silencing was measured by colony formation assay. (**G**). Statistical analysis of colony formation assay in DLD-1 and SW480 cells following TROP2 silencing. All of the experiments were performed in triplicate, and relative colony numbers are shown as means ± SD; ***P* ≤0.01, ****P*≤0.001. (**H**). Cell migration of DLD-1 and SW480 cells following TROP2 silencing migrating from serum-free to 20%FBS chambers. (**I**). Quantification of migrated cells; ***P* ≤0.01, ****P* ≤0.001, *****P* ≤0.0001. (**J**). In vitro invasion of DLD-1 and SW480 cells following TROP2 silencing. (**K**). Quantification of invasive cells; ***P* ≤0.01, ****P* ≤0.001. (**L**). Cell migration of DLD-1 and SW480 cells ± TROP2 OE migrating from serum-free to 20% FBS chambers. (**M**). Quantification of migrated cells; ***P* ≤0.01. (**N**). Photographic comparison of tumor sizes between groups. (**O**). Growth curves of tumors induced by specified cells. (**P**). Tumor weights in the specified groups.

**
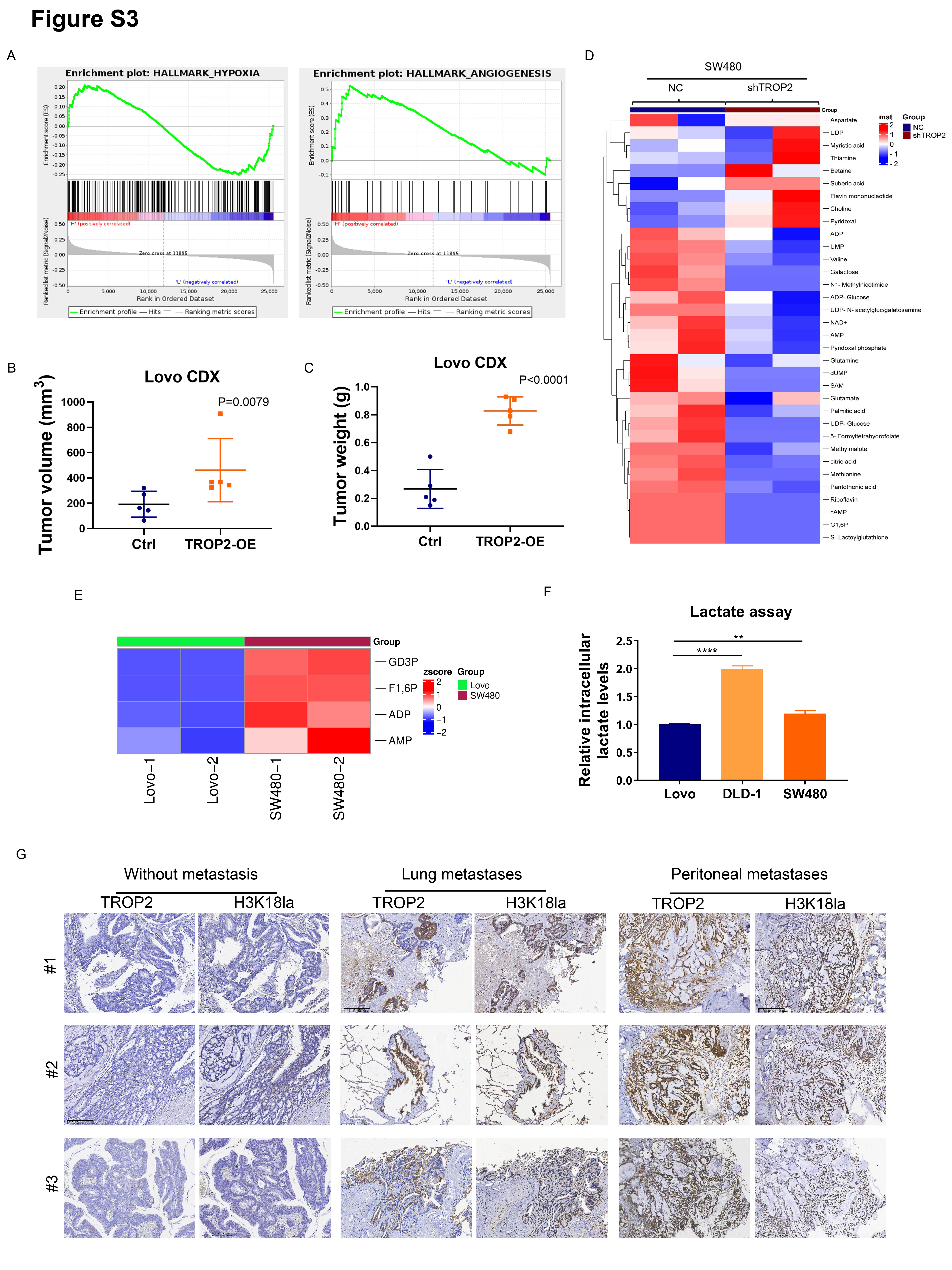
**

**Figure S3**. H3K18 lactylation is increased in CRC cells in response to TROP2 expression level. (**A**). GSEA analysis demonstrating correlation of high TROP2 expression with Hallmark Hypoxia (left), Angiogenesis (right) in CRC tissues, based on tissue microarray detection. (n=30; High, n = 15; Low, n = 15; based on median expression). (**B**). Tumor volumes in the specified groups. (**C**). Tumor weights in the specified groups. (**D**). Metabolomic comparison between NC and shTROP2 in SW480 adherent cells cultured in vitro. Two biological replicates are shown as separate columns for each cell type. (**E**). Metabolomic comparison between Lovo and SW480 cell lines. (**F**). Abundance of intracellular lactate in Lovo, DLD-1 and SW480 cells. ***P* ≤0.01, *****P* ≤0.0001. (**G**). Representative cases demonstrating high versus low TROP2/H3K18la expression in lung and peritoneal metastases and primary tumors from CRC patients without metastasis (n=9). (scale bar: 200 μm;)

**
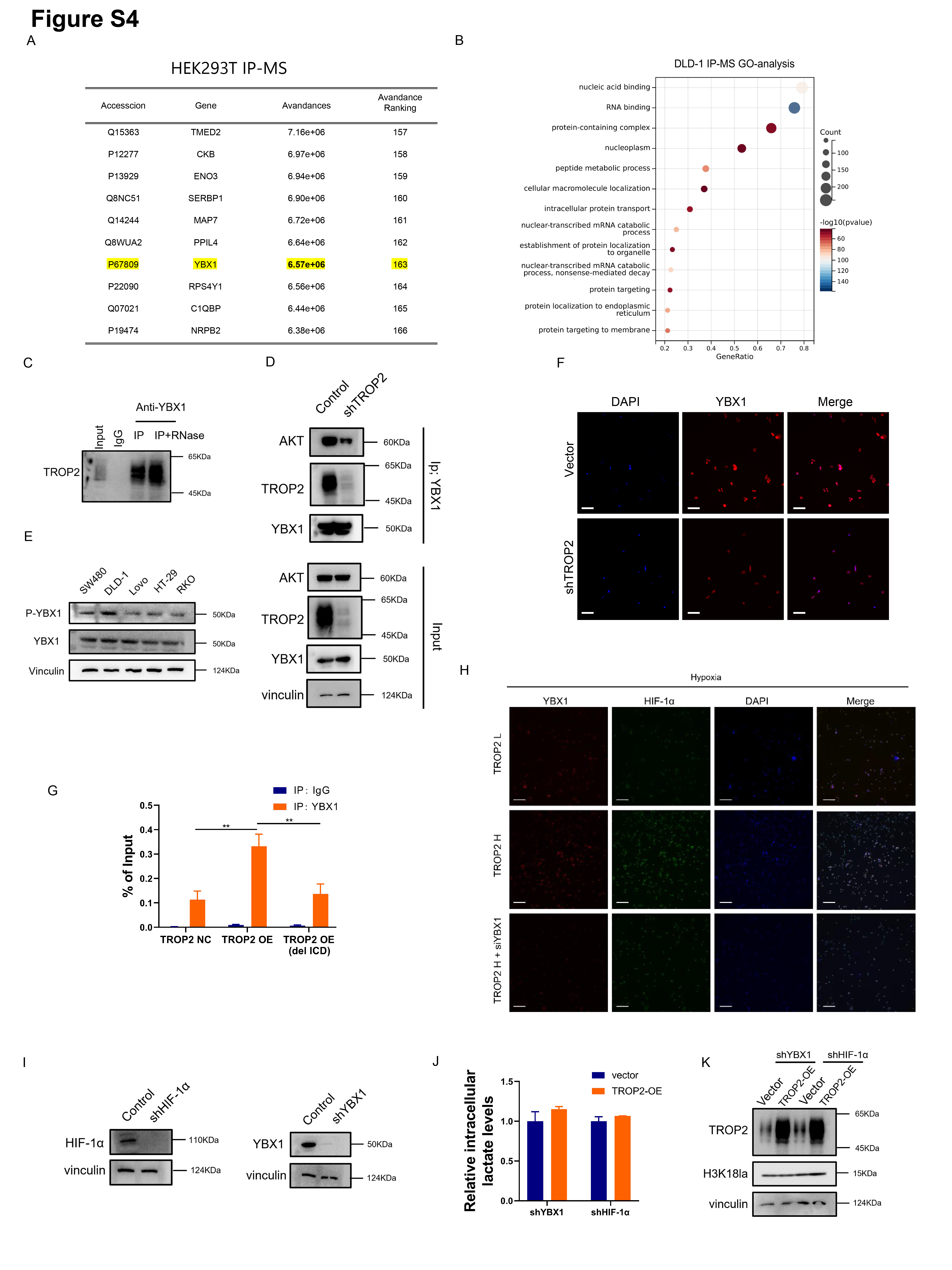
**

**Figure S4**. TROP2 promotes lactate production in CRC through YBX1-HIF-1α signaling (**A**). Table of potential TROP2-interacting proteins identified by IP-MS in the intracellular environment of HEK293 normal cells (listed in the order of abundance). (**B**). GO enrichment analysis of TROP2-interacting proteins identified by IP-MS in DLD-1 cells, revealing potential signaling pathways involving TROP2 in Colorectal Cancer (CRC). (**C**). Equal amounts of lysate were incubated with or without RNase and then subjected to immunoprecipitation using an anti-YBX1 antibody. (**D**). Endogenous AKT were IP from cells with Vector, shTROP2 using anti-YBX1 antibody, and then they were analyzed by western blot. (**E**). Western blotting detection of p-YBX1 in a group of CRC cell lines. (**F**). Images showing changes in the nuclear-cytoplasmic distribution of YBX1 (red) in SW480 cells following TROP2 silencing. (**G**). Immunoprecipitation of DNA fragments (ChIP) from SW480 cells transfected vector, TROP2, TROP2(del ICD) using an YBX1 specific antibody, followed by qPCR analysis with indicated primers; ***P* ≤0.01. (**H**). Immunofluorescence staining showing that TROP2 overexpression enhanced while siYBX1 reduced the protein levels of HIF-1α (green) in hypoxia; (scale bar: 100 μm). (**I**). Western blotting detection of YBX1 and HIF-1α in shYBX1 and shHIF-1α CRC cells. (**J and K**). shYBX1 and shHIF-1α CRC cells were transfected with TROP2 overexpression vector and then their intracellular lactate and H3K18la levels was dectected.


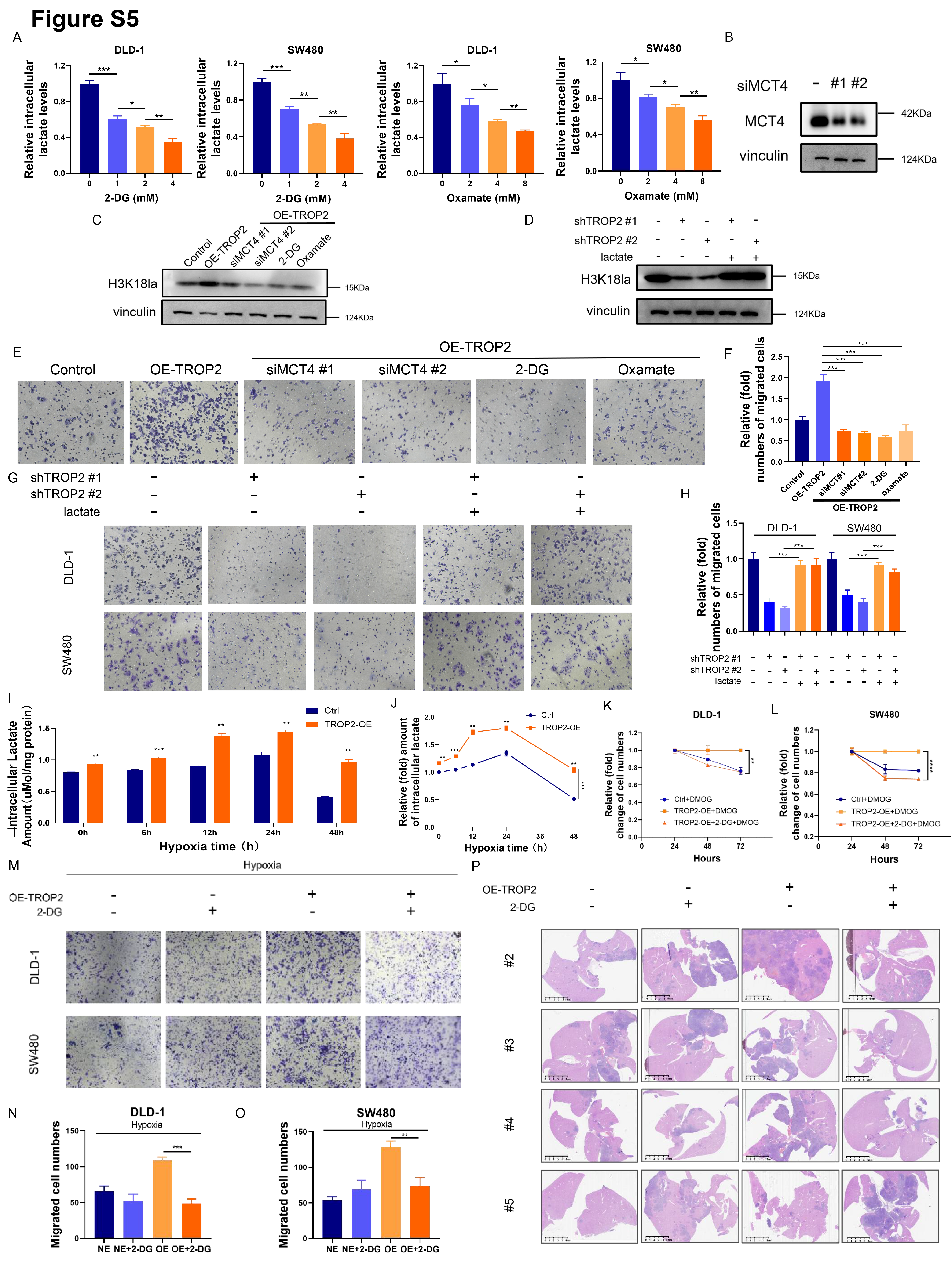


**Figure S5**. Inhibition of H3K18la inhibits TROP2-mediated CRC liver metastatic progression. (**A**). Abundance of intracellular lactate in DLD-1 and SW480 cells cultured in gradient concentrations of 2-DG or oxamate as measured by a lactate colorimetric kit, normalized to abundance in the 0mM group. Statistical significance was determined using one-way ANOVA followed by Sidak’s multiple comparisons test; **P* ≤0.05, ***P* ≤0.01, ****P* ≤0.001. (**B**). Western blotting detection of MCT4 in CRC cells transfected with MCT4 siRNAs. (**C** and **D**). Western blotting detection of H3K18la in TROP2-OE CRC cells treated by siRNA for MCT4, 2-DG, oxamate (C) and shTROP2 CRC cells treated by exogenous lactate (D). (**E**). Cell migration changes of TROP2-OE SW480 cells treated by siRNA for MCT4, 2-DG or oxamate. (**F**). Quantification of migrated cells; ****P* ≤0.001. (**G**). Cell migration changes of shTROP2 DLD-1 and SW480 cells treated by exogenous lactate. (**H**). Quantification of migrated cells; ****P* ≤0.001. (**I**). Abundance of intracellular lactate in control (Ctrl) and TROP2-OE SW480 cells exposed to hypoxia (1% oxygen) at indicated time points before harvest as measured by a lactate colorimetric kit, normalized to abundance in the Ctrl group at 0h. (For each time point, n=3, Mean ± SEM; Statistical significance was determined using t test; ***P* ≤0.01, ****P* ≤0.001). (**J**). Comparison of lactate accumulation kinetics of Ctrl and TROP2-OE SW480 cells exposed to hypoxia for indicated time. Statistical significance was determined using two-way ANOVA followed by Sidak’s multiple comparisons test; ; ***P* ≤0.01, ****P* ≤0.001. (**K** and **L**). Proliferation changes in TROP2-overexpressing DLD-1 (K) and SW480 (L) cells cultured in 2mM DMOG ± 2mM 2-DG were evaluated by CCK8 assay; **P* ≤0.05, *****P* ≤0.0001; OE, overexpression. (**M**). Cell migration changes of TROP2-OE DLD-1 and SW480 cells cultured in 2mM 2-DG under hypoxic conditions (1% oxygen), migrating from serum-free to 20%FBS chambers, compared to cells with normal TROP2 expression. (**N** and **O**). Quantification of migrated cells under hypoxic conditions (1% oxygen); ****P* ≤0.001. (**P**). Representative H&E staining images of liver metastasis in four additional (#2-5) mice per group after splenic injection of TROP2-OE MC38 cells, compared to cells with normal TROP2 expression, under 2-DG treatment (scale bar: 5mm).


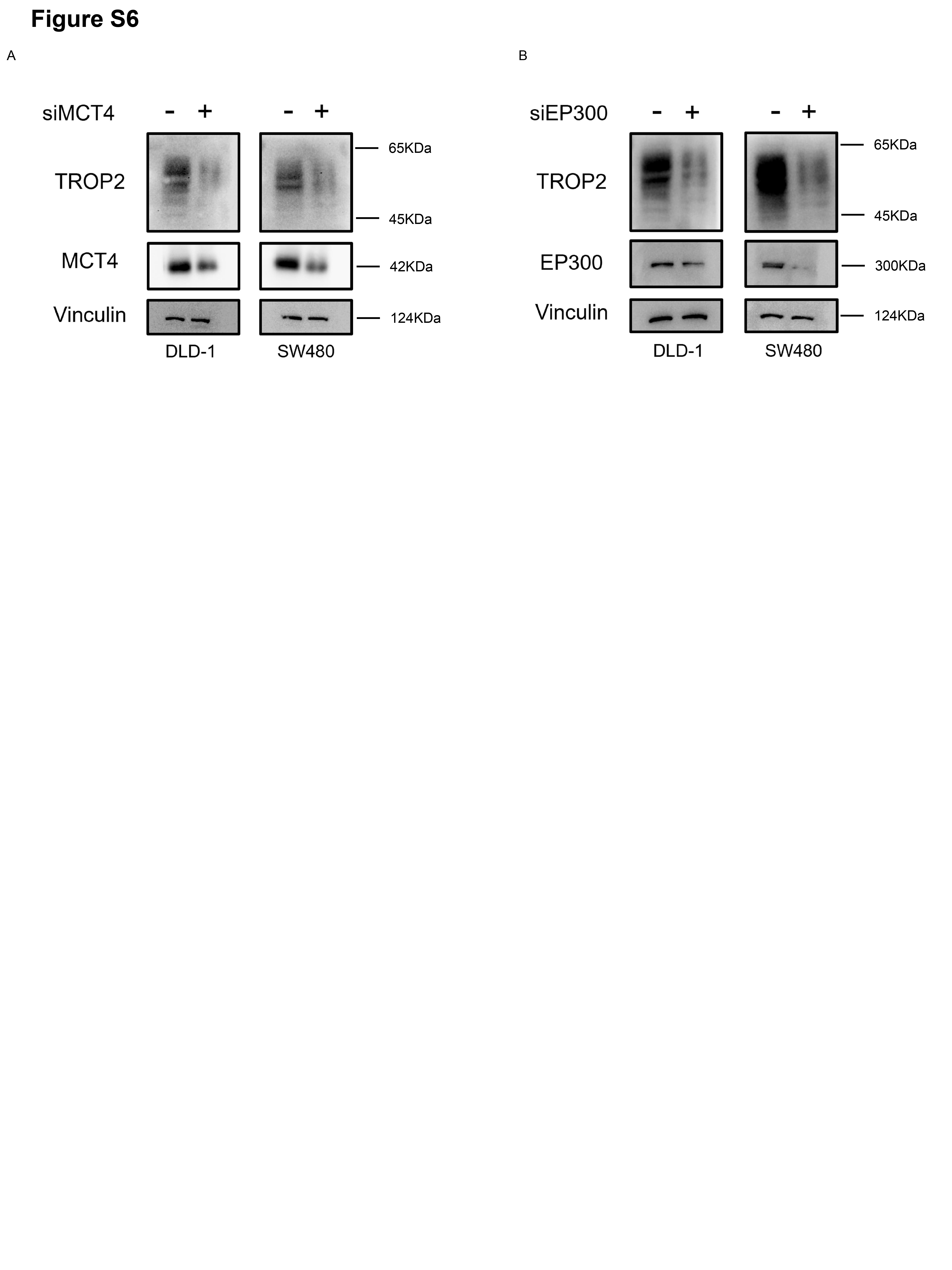


**Figure S6**. Genome-wide analysis of the transcriptional consequences of H3K18la in TROP2-high colorectal cancer. (**A** and **B**). Western blotting detection of TROP2 in DLD-1 and SW480 cells transfected with MCT4 (A) and EP300 (B) siRNA.


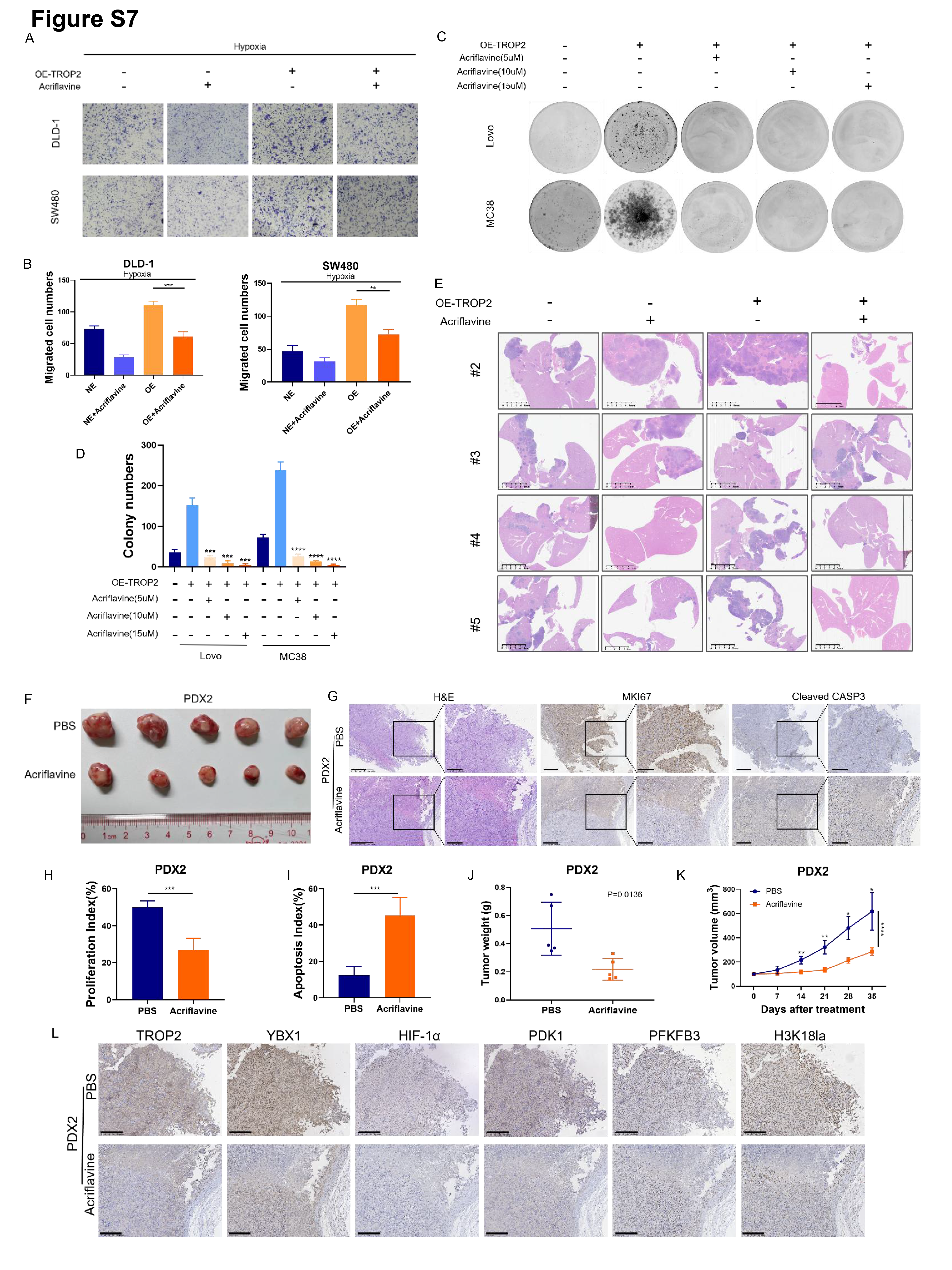


**Figure S7.** Acriflavine suppresses TROP2-driven CRLM progression by targeting the TROP2/YBX1/HIF-1α/H3K18la feeaback loop. (**A**). Migration changes of TROP2-OE DLD-1 and SW480 cells cultured in 10 μM Acriflavine under hypoxic conditions (1% oxygen), migrating from serum-free to 20%FBS chambers, compared to cells with normal TROP2 expression. (**B**). Quantification of migrated cells under hypoxic conditions (1% oxygen); ***P* ≤0.01, ****P* ≤0.001. (**C**). Tumorgenicity changes of TROP2-OE Lovo, MC38 cells treated with gradient concentrations of acriflavine were evaluated by colony formation assay and compared to cells with normal TROP2 expression. (**D**). Statistic analysis of colony formation assay in TROP2-OE Lovo, MC38 cells treated with gradient concentrations of acriflavine; All of the experiments were performed in triplicate, and relative colony numbers are shown as means ± SD; ****P* ≤0.001, *****P* ≤0.0001. (**E**). Representative H&E staining images of liver metastasis in four additional (#2-5) mice per group after splenic injection of TROP2-OE MC38 cells, compared to cells with normal TROP2 expression, under Acriflavine treatment (scale bar: 5mm). (**F**). Photographic comparison of patient-derived xenograft #2 (PDX2) tumors with PBS or acriflavine treatment. (**G**). Representative images of H&E and IHC staining for MKI67 or cleaved CASP3 in PDX2 tumors. (scale bar: left, 400 μm; right, 200 μm;). (**H** and **I**). Quantification of proliferation index (H) and apoptosis index (I) between groups for PDX2. (**J**). PDX2 tumor weights between groups. (**K**). Growth curves of PDX2 tumors between groups. (**L**). Representative paraffin-embedded sections of PDX2 tumors for each group stained with antibodies against TROP2, YBX1, HIF-1α, PDK1, PFKFB3, H3K18la; (scale bar: 200 μm).


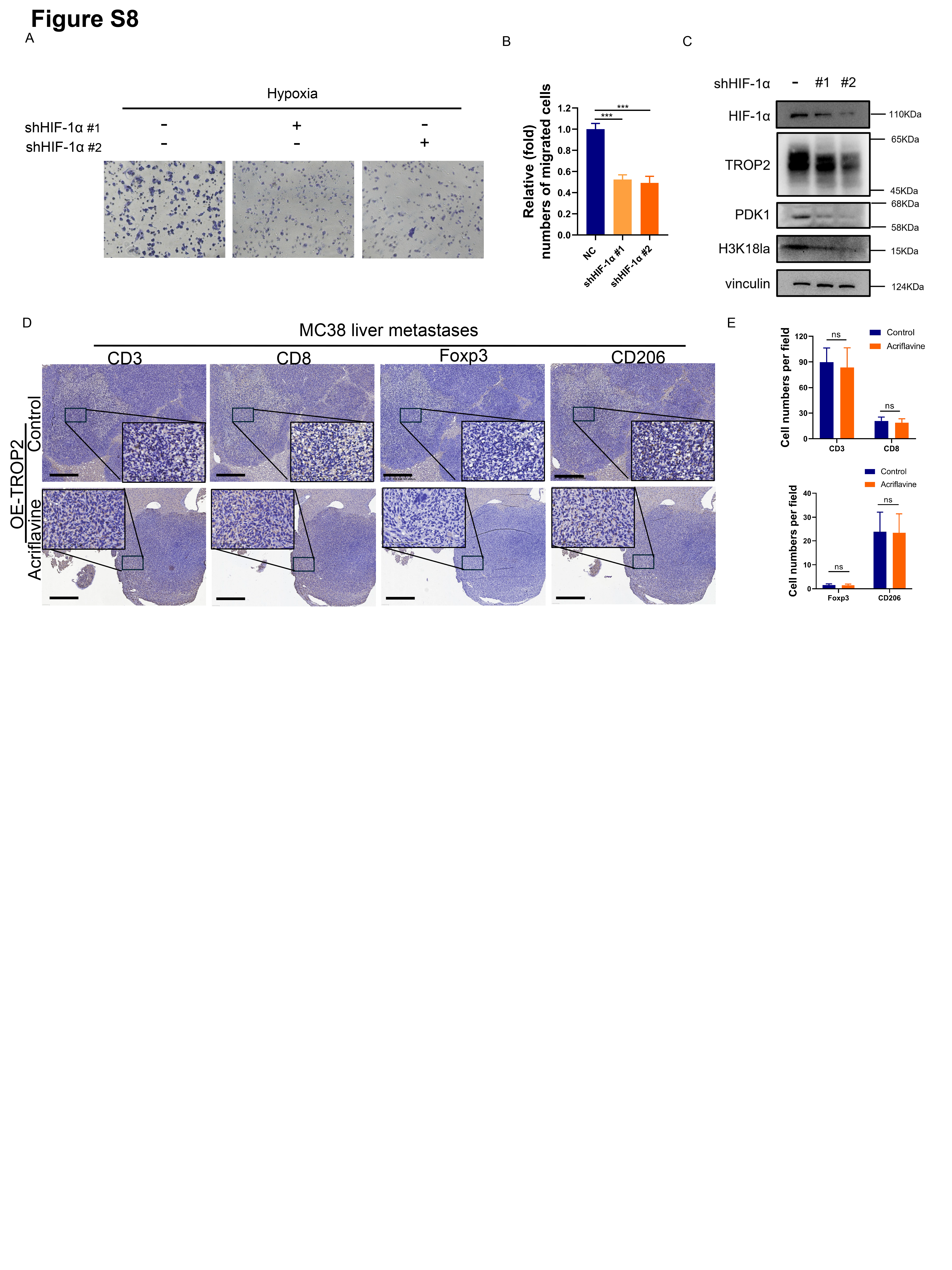


**Figure S8.** Acriflavine suppresses TROP2-driven CRLM progression by targeting the TROP2/YBX1/HIF-1α/H3K18la feeaback loop. (**A**). Migration changes of TROP2-high SW480 cells after HIF-1α knockdown under 1% O₂ hypoxia. (**B**). Quantification of migrated cells; ****P* ≤0.001. (**C**). Western blotting detection of TROP2/YBX1/HIF-1α/PDK1/H3K18la in SW480 cells after HIF-1α knockdown under normoxia. (**D**). Representative IHC images of CD3, CD8, Foxp3, CD206 in liver metastases from acriflavine-treated and control mice spleen injected with TROP2-OE MC38 cells. (scale bar: 400 μm;) (**E**). Comparison of CD3⁺, CD8⁺, Foxp3⁺ and CD206⁺ cell counts between both groups.


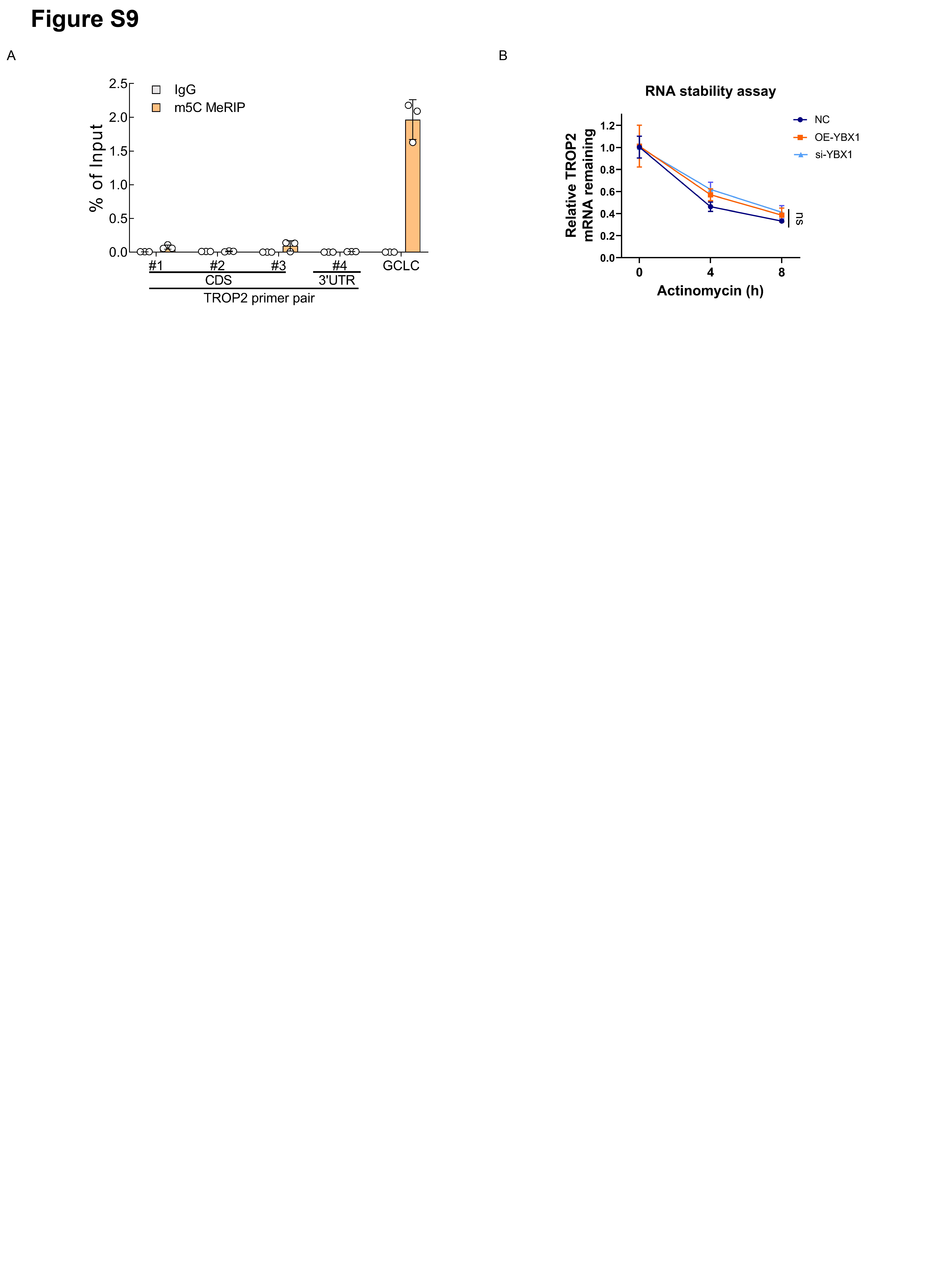


**Figure S9** (**A**). RNA fragments from SW480 cells were immunoprecipitated with the m5C specific antibody (m5C meRIP) and analyzed by qPCR using the indicated primers. GCLC mRNA is positive control; CDS：Coding Sequence; 3’ UTR：3’ Untranslated Region. (**B**). mRNA degradation curves of TROP2 over time in SW480 cells following YBX1 overexpression or silencing under the treatment of actinomycin D. Cells were cultured in complete medium.
