# Supplementary material for: Feedback regulation between histone H3 lysine 18 lactylation and TROP2‐mediated glycolysis drives metastatic progression of colorectal cancer
Source: Clin Transl Med. 2026 Jan 3;16(1):e70562. doi: 10.1002/ctm2.70562 (PMC12761367; doi:10.1002/ctm2.70562)
Supplement: Supplementary file 2 — Supporting Information [file CTM2-16-e70562-s006.docx]

**Supplemental Table S1. Antibodies, reagents, assays and models used in this study**

| **Antibodies/Regents/Assays/Models** | **Source** | **Catalog#** |
| --- | --- | --- |
| **Antibodies** |  |  |
| Anti-TROP2 | Abcam | Cat# ab214488; RRID:AB_2811182 |
| Anti-TROP2 | Cell Signaling Technology | Cat# 90540; RRID:AB_2800160 |
| Anti-TROP2 | Proteintect | Cat# 27360-1-AP; RRID:AB_2918122 |
| Anti-YBX1 | Abcam | Cat# ab76149; RRID:AB_2219276 |
| Anti-YBX1 | Proteintect | Cat#20339-1-AP;RRID:AB_10665424 |
| Anti-p-YBX1 | Cell Signaling Technology | Cat# 2900; RRID:AB_2219273 |
| Anti-PDK1 | Cell Signaling Technology | Cat# 3062; RRID:AB_2236832 |
| Anti-PFKFB3 | Cell Signaling Technology | Cat# 13123; RRID:AB_2617178 |
| Anti-PKM2 | Cell Signaling Technology | Cat# 4053; RRID:AB_1904096 |
| Anti-HIF-1α | Abcam | Cat# ab51608; RRID:AB_880418 |
| Anti-HIF-1α | Abcam | Cat# ab1; RRID:AB_296474 |
| Anti-Pan Kla | PTM BIO | Cat# PTM-1401; RRID: N/A |
| Anti-H3K18la | PTM BIO | Cat# PTM-1427RM; RRID: N/A |
| Anti-H3K9la | PTM BIO | Cat# PTM-1419RM; RRID: N/A |
| Anti-H4K5la | PTM BIO | Cat# PTM-1407RM; RRID: N/A |
| Anti-H4K8la | PTM BIO | Cat# PTM-1415; RRID: N/A |
| Anti-H4K12la | PTM BIO | Cat# PTM-1411; RRID: N/A |
| Anti-vinculin | Cell Signaling Technology | Cat# 4650; RRID:AB_10559207 |
| Anti-Lamin A | Cell Signaling Technology | Cat# 86846; RRID:AB_2800093 |
| Anti-LDHA | Cell Signaling Technology | Cat# 2012; RRID:AB_2137173 |
| Anti-FLAG tag (rabbit) | Cell Signaling Technology | Cat# 14793; RRID:AB_2572291 |
| Anti-FLAG tag (mouse) | Cell Signaling Technology | Cat# 8146; RRID:AB_10950495 |
| Anti-HA tag (rabbit) | Cell Signaling Technology | Cat# 3724; RRID:AB_1549585 |
| Anti-HA tag (mouse) | Cell Signaling Technology | Cat# 2367; RRID:AB_10691311 |
| HRP-conjugated secondary antibodies (anti-rabbit) | Cell Signaling Technology | Cat# 7074; RRID:AB_2099233 |
| HRP-conjugated secondary antibodies (anti-mouse) | Cell Signaling Technology | Cat# 7076; RRID:AB_330924 |
| Anti-MKI67 | proteintect | Cat# 27309-1-AP; RRID:AB_2756525 |
| Anti-Cleaved CASP3 | Cell Signaling Technology | Cat# 9661; RRID:AB_2341188 |
| IHC Secondary Antibodies | ZSGB-BIO | Cat# PV-6000; RRID: N/A |
| Fluorescent-tagged secondary antibodies | Abcam | Cat# ab150080; RRID:AB_2650602 |
| Fluorescent-tagged secondary antibodies | Abcam | Cat# ab150113; RRID:AB_2576208 |
| CD3 | proteintect | Cat#17617-1-AP; RRID:AB_1939430 |
| CD8 | proteintect | Cat#29896-1-AP; RRID:AB_2935485 |
| Foxp3 | proteintect | Cat#22228-1-AP; RRID:AB_11182376 |
| CD206 | proteintect | Cat#18704-1-AP; RRID:AB_10597232 |
| MCT4 | proteintect | Cat#22787-1-AP; RRID:AB_11182479 |
| EP300 | proteintect | Cat#20695-1-AP; RRID:AB_3085614 |
| **Regents** |  |  |
| shTROP2#1 lentiviral particles | Genecopoeia | Cat #LPP-HSH010940  –LVRU6rLP -b-200; RRID: N/A |
| shTROP2#2 lentiviral particles | Genecopoeia | Cat #LPP-HSH010940 -LVRU6rLP-c-200; RRID: N/A |
| shTROP2#3 lentiviral particles | Genecopoeia | Cat #LPP-HSH010940 -LVRU6rLP-a-200; RRID: N/A |
| Scrambled Control | Genecopoeia | Cat#LPP-CSHCTR001  -LVRU6Rlp-025-C; RRID: N/A |
| TROP2 overexpression lentiviral particles | Genecopoeia | Cat #LPP-G0457-Lv217  -100; RRID: N/A |
| Mouse: Trop2 overexpression lentiviral particles | Genecopoeia | Cat #LPP-Mm07769-Lv217  -100; RRID: N/A |
| Truncated plasmid (TROP2-FL) | Kidan Bio Co. Ltd. (Guangzhou, China) | N/A; RRID: N/A |
| Truncated plasmid (TROP2-del 1-275aa) | Kidan Bio Co. Ltd. (Guangzhou, China) | N/A; RRID: N/A |
| Truncated plasmid (TROP2-del 276-297aa) | Kidan Bio Co. Ltd. (Guangzhou, China) | N/A; RRID: N/A |
| Truncated plasmid (TROP2-del 297-323aa) | Kidan Bio Co. Ltd. (Guangzhou, China) | N/A; RRID: N/A |
| RIPA | Beyotime | P0013B; RRID: N/A |
| TRIzol Reagent | Invitrogen | 15596026; RRID: N/A |
| 2-DG | MedChemExpress | HY-13966; RRID: N/A |
| Oxamate | MedChemExpress | HY-W013032A; RRID: N/A |
| Acriflavine | MedChemExpress | HY-100575; RRID: N/A |
| Pierce Protein A/G Magnetic Beads | Invitrogen | 88802; RRID: N/A |
| Anti-HA Magnetic Beads | MedChemExpress | HY-K0201; RRID: N/A |
| Anti-Flag Magnetic Beads | MedChemExpress | HY-K0207; RRID: N/A |
| DMOG | MedChemExpress | HY-15893; RRID: N/A |
| Lactate | MedChemExpress | HY-B2227BR; RRID: N/A |
| PBS | Biosharp | BL-601A; RRID: N/A |
| Permeabilization buffer | eBioscience | 00-8333-56; RRID: N/A |
| DAPI | Solarbio | C0065; RRID: N/A |
| Puromycin | Solarbio | P8230; RRID: N/A |
| jetPRIME® transfection reagent | Polyplus | 101000046; RRID: N/A |
| Protease and phosphatase inhibitor cocktail | Thermo Fisher | 78446; RRID: N/A |
| siRNA#1 targeting human YBX1 (target sequence:5’-GGAACGGAUAUGGUUUCAUTT-3') | (Chen, Li et al. 2019) | N/A; RRID: N/A |
| siRNA#2 targeting human YBX1 (target sequence:5'-GGUUCCCACCUUACUACAUTT-3') | (Chen, Li et al. 2019) | N/A; RRID: N/A |
| **Commercial assays** |  |  |
| Human Transcriptome Array (HTA) 2.0 arrays | Thermo Fisher | N/A; RRID: N/A |
| PierceTM BCA Protein Assay Kit | Thermo Fisher | 23227; RRID: N/A |
| Lactate Assay Kit-WST | DOJINDO | L256; RRID: N/A |
| Seahorse XF Glycolysis Stress Test Kit | Seahorse | 103020; RRID: N/A |
| Seahorse XF Cell Mito Stress Test Kit | Seahorse | 103015; RRID: N/A |
| Pierce™ Magnetic ChIP Kit | Thermo Fisher | 26157; RRID: N/A |
| PrimeScript RT Kit | TaKaRa Bio | RR037A; RRID: N/A |
| SYBR Green PCR Master Mix | Life Technologies | 4367659; RRID: N/A |
| Cell Counting Kit-8 | Dojindo | CK04; RRID: N/A |
| H&E staining Kit | Solarbio | G1120; RRID: N/A |
| Pierce™ Classic Magnetic IP/Co-IP Kit | Thermo Fisher | 88804; RRID: N/A |
| Immunohistochemistry Kit | Cell biological | CEK5007; RRID: N/A |
| **Cell lines** |  |  |
| HEK293T | ATCC | CRL-3216; RRID:CVCL_0063 |
| CCD-841CoN | ATCC | CRL-1790; RRID:CVCL_2871 |
| Lovo | ATCC | CCL-229; RRID:CVCL_0399 |
| DLD-1 | ATCC | CCL-221; RRID:CVCL_0248 |
| SW480 | ATCC | CCL-228; RRID:CVCL_0546 |
| HT-29 | ATCC | HTB-38; RRID:CVCL_0320 |
| HCT-116 | ATCC | CCL-247; RRID:CVCL_0291 |
| RKO | ATCC | CRL-2577; RRID:CVCL_0504 |
| SW620 | ATCC | CCL-227; RRID:CVCL_0547 |
| CaCO2 | ATCC | HTB-37; RRID:CVCL_0025 |
| Mouse:MC-38 | Kerafast | ENH204-FP; RRID:CVCL_B288 |
| **Mice** |  |  |
| Mouse: C57BL/6J | GemPharmatech, China | N000013; RRID: N/A |
| BALB/c-Nude | GemPharmatech, China | D000521; RRID: N/A |
| NOD-Scid | GemPharmatech, China | T001492; RRID: N/A |

**Reference**

Chen, X., et al. (2019). "5-methylcytosine promotes pathogenesis of bladder cancer through stabilizing mRNAs." Nat Cell Biol **21**(8): 978-990.
