# Supplementary material for: Feedback regulation between histone H3 lysine 18 lactylation and TROP2‐mediated glycolysis drives metastatic progression of colorectal cancer
Source: Clin Transl Med. 2026 Jan 3;16(1):e70562. doi: 10.1002/ctm2.70562 (PMC12761367; doi:10.1002/ctm2.70562)
Supplement: Supplementary file 6 — Supporting Information [file CTM2-16-e70562-s003.docx]

**Supplemental Table S5. Primers used in this study.**

**RT-qPCR primers:**

| **Gene** | **Sequences (5’-3’)** |
| --- | --- |
| HK2-Forward | GATTGTCCGTAACATTCTCATCG |
| HK2-Reverse | CAGGCAGTCACTCTCAATCTGAG |
| PFKFB3-Forward | CTCGCATCAACAGCTTTGAGG |
| PFKFB3-Reverse | TCAGTGTTTCCTGGAGGAGTC |
| ALDOA-Forward | CAGGGACAAATGGCGAGACTA |
| ALDOA-Reverse | GGGGTGTGTTCCCCAATCTT |
| ENO2-Forward | CCGGGAACTCAGACCTCATC |
| EN02-Reverse | CTCTGCACCTAGTCGCATGG |
| PKM2-Forward | ATAACGCCTACATGGAAAAGTGT |
| PKM2-Reverse | TAAGCCCATCATCCACGTAGA |
| LDHA-Forward | ATGGCAACTCTAAAGGATCAGC |
| LDHA-Reverse | CCAACCCCAACAACTGTAATCT |
| PIK3CB-Forward | TATTTGGACTTTGCGACAAGACT |
| PIK3CB-Reverse | TCGAACGTACTGGTCTGGATAG |
| SOS2-Forward | CCGCAGCCTTACGAGTTCTTC |
| SOS2-Reverse | GGATGCACTTGTTCCTGAACC |
| MAP4K4-Forward | GACTCCCCTGCAAAAAGTCTG |
| MAP4K4-Reverse | GTCCATAGGTGCCATTTCCAA |
| POSTN-Forward | CTCATAGTCGTATCAGGGGTCG |
| POSTN-Reverse | ACACAGTCGTTTTCTGTCCAC |

**ChIP-qPCR primers:**

| **Gene** | **Sequences (5’-3’)** |
| --- | --- |
| HIF1A-Forward | CGCCACCAACAATACTAAG |
| HIF1A-Reverse | TGGGAAATGCCTGAAGAG |
| TROP2-Forward | GACATTGCCCGGAAACTCAG |
| TROP2-Reverse | GGGACTTTTCACAAATGCCGA |

**m5C meRIP-qPCR primers:**

| **Gene** | **Sequences (5’-3’)** |
| --- | --- |
| TROP2-Forward (CDS) | ACAACGATGGCCTCTACGAC |
| TROP2-Reverse (CDS) | GTCCAGGTCTGAGTGGTTGAA |
| TROP2-Forward (CDS) | CGGCAGAACACGTCTCAGAAG |
| TROP2-Reverse (CDS) | CCTTGATGTCCCTCTCGAAGTAG |
| TROP2-Forward (CDS) | CATCAAGGGCGAGTCTCTATTC |
| TROP2-Reverse (CDS) | CCCGACTTTCTCCGGTTGG |
| TROP2-Forward (3’UTR) | TTCGGTCCAACAACAGGAAACC |
| TROP2-Reverse (3’UTR) | GAACTCATCAAATAATTTAAACCCAAG |
| GCLC-Forward | AAAAGTCCGGTTGGTCCTG |
| GCLC-Reverse | CCTGGTGTCCCTTCAATCATG |
